# Deep reinforcement learning for resource allocation and scalable numerology in NR-U enabled multi-RAT HetNets

**DOI:** 10.1038/s41598-026-36539-6

**Published:** 2026-02-03

**Authors:** Noha A. Elmosilhy, Mahmoud M. Elmesalawy, Ahmed M. Abd El-Haleem, Ibrahim I. Ibrahim

**Affiliations:** 1 Department of Communications and Electronics, Canadian International College, CIC, Cairo, 11865 Egypt; 2 Department of Electronics, Communications and Computer, Faculty of Engineering, Helwan University, Cairo, 11722 Egypt

**Keywords:** HetNet, NR-U, Multi-RAT, Network slicing, Numerology, Regret matching, Multiple radio access technologies (Multi-RAT), Heterogeneous network (HetNet), Deep reinforcement learning (DRL), Engineering, Mathematics and computing

## Abstract

Leveraging the new radio technology in the unlicensed band (NR-U) can alleviate traffic congestion, enhance network capacity, and help mitigate the diversity in users’ service requests. In this paper, a multiple slice multi-radio access technology (RAT) heterogeneous network (HetNet) is considered, integrating the new radio (NR) technology in the licensed and unlicensed bands. An optimization problem is proposed aiming to maximize users’ satisfaction, defined by maximizing the achievable data rate while maintaining the minimum latency slice requirement. To solve the proposed optimization problem, an iterative framework is introduced that utilizes deep reinforcement learning (DRL) algorithm jointly with the regret learning algorithm (RLA) that efficiently solves users’ association problem considering coexisting Wi-Fi users, allocates radio resources for each slice and determines the optimum scalable numerology value in each slice. The simulation results show that our proposed model improves users’ satisfaction, achieving up to 70% user satisfaction compared with other baseline approaches.

## Introduction

The increasing integration of Machine Learning (ML) and Internet of Things (IoT) is creating the expectation that Beyond Fifth Generation (B5G) communications will fulfill diverse Quality of Service (QoS) requirements across a wide range of mission-critical applications. These mission-critical applications include Extended Reality (XR), Vehicle-to-Everything (V2X), industrial automation and tactile internet. The New Radio (NR) standard categorizes these applications based on their varying requirements into services such as Ultra-Reliable Low Latency Communication (URLLC), Enhanced Mobile Broadband (eMBB) and Massive Machine Type Communication (MTC)^1^. Consequently, Network Slicing (NS) can be beneficial in supporting these distinct services, allowing for effective fulfillment of QoS needs^2^. NS technology has been introduced to enhance network efficiency. An End-to-End (E2E) network slice is segmented into sub-network slices, including the Radio Access Network (RAN), transport, and Core Network (CN) layers, connecting end-user devices, leveraging Software-Defined Networks (SDN) and Network Function Virtualization (NFV)^3^. NS enables the creation of multiple logical networks, each tailored to support specific services with distinct requirements.

These logically independent networks are referred to as RAN slices. For instance, a RAN slice dedicated to URLLC must ensure very low latency and high reliability; conversely, a slice designed for eMBB needs to provide high data rates to accommodate services such as video streaming and virtual reality. The significant challenge lies in meeting the QoS requirements for each slice, given the diverse needs and the limited resources of the existing physical infrastructure. Each Base Station (BS) can host several slices, each serving different services and users, necessitating an effective resource allocation mechanism. This mechanism must manage the allocation of Resource Blocks (RBs) efficiently across these slices to address the varying demands of heterogeneous users and services. Developing a robust resource slicing framework to meet diverse services’ requirements remains a major challenge in B5G and 6G networks.

Moreover, to support the diversity in each service’s requirements, 3rd Generation Partnership Project (3GPP) standardized scalable numerology and mini-slot approach^4^, promoting the adaptability of the physical layer in NR. Unlike Long Term Evolution (LTE), which is limited to a single numerology in the downlink and fixed slot duration, the introduction of mini-slots allows rapid transmission of low-latency data without waiting for slot boundaries. Although the scalable numerology technique accommodates various subcarrier spacing, symbol durations, however, a significant challenge is initiated in optimizing the scalable numerology value for each slice to effectively address the scheduling needs of both eMBB and URLLC users in Multi-user environments.

Furthermore, developing an efficient approach to address the co-existence problem between URLLC and eMBB services, a global optimal solution is needed. Thus, in this work, a Reinforcement Learning (RL) algorithm is required to obtain the most efficient scalable numerology value and effectively allocate RBs in each slice. RL algorithm works effectively for solving unknown stochastic dynamic situations with state spaces that are either continuously distributed or noticeably large^5,6^.

In addition, with the finite resources of 6G NR and with the growth of users’ service demands, 6G NR may fail to satisfy each user’s service requirement. Therefore, exploiting the unlicensed band to manage and improve the 6G NR limited capacity may present a promising solution for maintaining the required QoS across various network slices. The 3GPP Release 12 was the first attempt to introduce LTE to the 5GHz unlicensed band (LTE-U), followed by License-Assisted Access (LAA) in 3GPP Release 13. Successor to LAA, the 3GPP standardized New Radio Unlicensed (NR-U) standard^7,8^. The first attempt was in Release 16^9^, adopting the same LAA features, while in 3GPP Release 17, the NR-U outperforms the LAA due to the addition of scalable numerology features, which extend URLLC capabilities into the unlicensed spectrum.

The 5G NR-U can operate in three different scenarios^10^. The first scenario features Carrier Aggregation (CA) technology between a licensed band NR small cell and an unlicensed band NR-U small cell. In the second scenario, the Dual Connectivity (DU) technology is used between a licensed band LTE small cell and an unlicensed band NR-U small cell. The third scenario relies solely on the stand-alone unlicensed band NR-U. CA technology standardized in 3GPP Rel-10 is considered a very promising feature for high-rate data services.

Moreover, in CA-based multiple Radio Access Technology (RAT) Heterogeneous Network (HetNet), a user who can access both NR and NR-U RATs can choose between three different transmission modes, which can be defined as NR-mode, where a user can associate with NR-Small Cell (NR-SC) using NR RAT, or NR-U mode, where a user can associate with NR-U SC accessing the unlicensed band, or CA mode, where a user can associate with both NR SC and NR-U SC. Consequently, a new user association approach needs to be developed to efficiently associate users with the appropriate SC that can ensure the user’s Service Level Agreement (SLA) for the requested service.

Based on this, a Regret Learning Algorithm (RLA) is used in this paper to efficiently associate users with proper SC that can fulfill each user’s requested service requirement in a Multi-RAT HetNet network architecture, considering CA technology.

Motivated by the previous observation and to overcome the aforementioned challenges, this paper proposes an approach of deploying a Multi-RAT HetNet, leveraging the aggregation between the NR-U and NR licensed band, exploiting 6G NS and scalable numerology technology, aiming to maintain each user’s satisfaction, which is defined in achieving the SLA of each user’s requested service. Specifically, the contribution of this work is summarized as follows:

1. To the best of our knowledge, an architecture that integrates NR-U technology with network slicing for eMBB and URLLC services in multi-RAT HetNet, while supporting flexible numerology and considering WiFi co-existence, has not been studied. Therefore, a dynamic radio resource allocation scheme is studied in Multi RAT HetNet consists of one NR Macro Base Station (MBS) and several small cells. The small cells support three different transmission modes (NR mode, NR-U mode, and CA aggregation mode).
2. Since applying network slicing creates diversity in users’ requirements, a user association problem is formulated that maximizes individual user satisfaction. To quantify each user’s satisfaction, a satisfaction index is formulated in terms of achievable downlink data rate and achievable delay, taking into consideration each user’s service request.
3. The optimization problem is formulated to dynamically allocate slice resources, expressed as the slice ratio, and select the most appropriate scalable numerology value for each slice. To solve this, a multi-agent DRL algorithm is developed that continuously adapts the slice ratio while also determining the best numerology configuration for every slice.
4. A regret matching learning approach is also adopted that effectively manages user association in a multi-mode multi-RAT HetNet, guided by a unified utility function that captures user satisfaction. To the best of our knowledge, this is the first study to apply regret learning within an integrated network architecture that incorporates NR-U technology.
5. We proposed and analyzed a mathematical model to study delay in the CA mode, which has not been previously addressed in existing literature. Our model provides a comprehensive framework for understanding the impact of various parameters on delay performance on both NR and NR-U.
6. The performance of the proposed algorithm is thoroughly evaluated through detailed comparisons with several benchmark scenarios. The study covers different RAT deployments, including LTE-only, LTE–WiFi, and LWA, and considers both cases with and without WiFi coexistence. In addition, the proposed DRL method is compared with other reinforcement learning techniques to highlight its performance advantages.

The rest of the paper is organized as follows. Section II presents the literature review. Section III provides a detailed description of the proposed system design and problem formulation, in addition to the table of notation Table 1. The system model is formulated in Section IV. Section V introduces the channel access scheme. Section VI presents the NR and NR-U rate and delay analysis. Section VII introduces the proposed model framework. Section VIII presents the simulation results, providing a detailed analysis and evaluation of the proposed algorithms. Finally, Section IX concludes the paper.

## Literature review

Adopting RAN slicing in a Multi-RAT HetNet introduces challenges in resource allocation, as it dictates how physical resources within each SC can be partitioned among various slices. Several approaches have been proposed in the literature, exploring various schemes and algorithms to address this challenge^11,12^.

Furthermore, adopting the 5G NR standard scalable numerology technique may allow fulfilling the diversity of each service SLA. However, it will introduce a new challenge in efficiently choosing the appropriate scalable numerology value for each service. In^13^, the authors proposed a multi-cell, multi-timescale framework that exploits numerology technique within a network slicing architecture, combined with a parallel hierarchical DRL algorithm, to enhance resource utilization while jointly satisfying QoS requirements. Paper^14^ proposed a numerology-based resource allocation scheme that examines the impact of URLLC users on eMBB users. The proposed algorithm is formulated as a mixed-integer non-linear problem. while in^15^, a one–to–one matching game is introduced to optimize the lowest possible data rate for eMBB users, while applying NR scalable numerology approach to serve users who are demanding URLLC services.

All the aforementioned studies tend to strike a balance of providing an optimal resource allocation scheme that can utilize the available resources more effectively, along with meeting the QoS requirements of the co-existing slices. To achieve a global optimum solution for resource allocation across multiple slices in a network, while determining an optimal scalable numerology value for each slice that effectively meets each user’s SLA, many studies have proposed RL to solve the resource allocation problem among multiple slices. As in^16^, the authors developed a scalable resource allocation scheme based on multiple numerologies utilizing deep reinforcement learning to maximize each user’s Quality of Experience (QoE) constraints. In addition, in^17^, a RAN slicing problem is formulated and addressed using a hierarchical reinforcement learning framework. The authors in^17^ deployed an NR scalable numerology technique and mini-slot-based transmission to support both eMBB and URLLC network slices. In^18^, the authors proposed a DRL-based resource allocation framework for network slicing in RANs that leverages Massive Multiple-Input Multiple-Output (MIMO) technology. The framework aims to improve resource allocation efficiency across slices while enhancing user quality of experience.

The above studies focus on resource allocation to support the diverse needs of URLLC and eMBB services. However, under heavy load with many users and varied requests, the limited capacity of the 5G NR licensed band remains a challenge, even with network slicing and scalable numerology. While slicing helps meet user SLAs, the licensed band alone can still suffer from congestion and performance loss. Using the unlicensed band is therefore a promising way to ease these limits and maintain QoS across different slices.

Many studies have explored extending NR capacity by using the unlicensed band alongside WiFi.In^19^, a comparison between WiFi and NR-U channel access mechanisms is studied. In^20^, the authors proposed a network slicing scheme with scalable numerology that integrates LTE-WLAN Aggregation (LWA) to overcome the limits of licensed spectrum while maintaining user SLAs. Their results show improved user satisfaction with the proposed multi-RAT HetNet architecture, but the slicing and scalable numerology were applied only in the licensed band.

Based on this, exploiting both the 5G NR licensed and unlicensed bands can help overcome the limited capacity of congested NR networks. However, the use of NR-U introduces several challenges, such as ensuring fair coexistence with WiFi, and many studies have proposed schemes to address this channel-sharing problem. In^21^, the authors studied how different configurations of switching patterns and RLC timing parameters affect NR-U performance in the presence of WiFi. The results highlight both the advantages and limitations of NR-U, with a focus on its coexistence with WiFi. while authors in In^22^, the authors presented a machine learning data-driven approach for spectrum sharing between NR-U and Wi-Fi. The paper investigated a gap-based method integrated with the LBT back-off process and analyzed its impact on the spectrum utilization of the unlicensed band. while authors in^23^ introduced a DRL approach to dynamically optimize the Energy Detection (ED) values in NR-U and WiFi coexistence for URLLC downlink transmission.

On the other hand, relying only on offloading users’ requests to the unlicensed band with NR-U cannot fully meet user demands in congested networks. Aggregating both licensed and unlicensed bands can provide higher bandwidth,

**Fig. 1:**
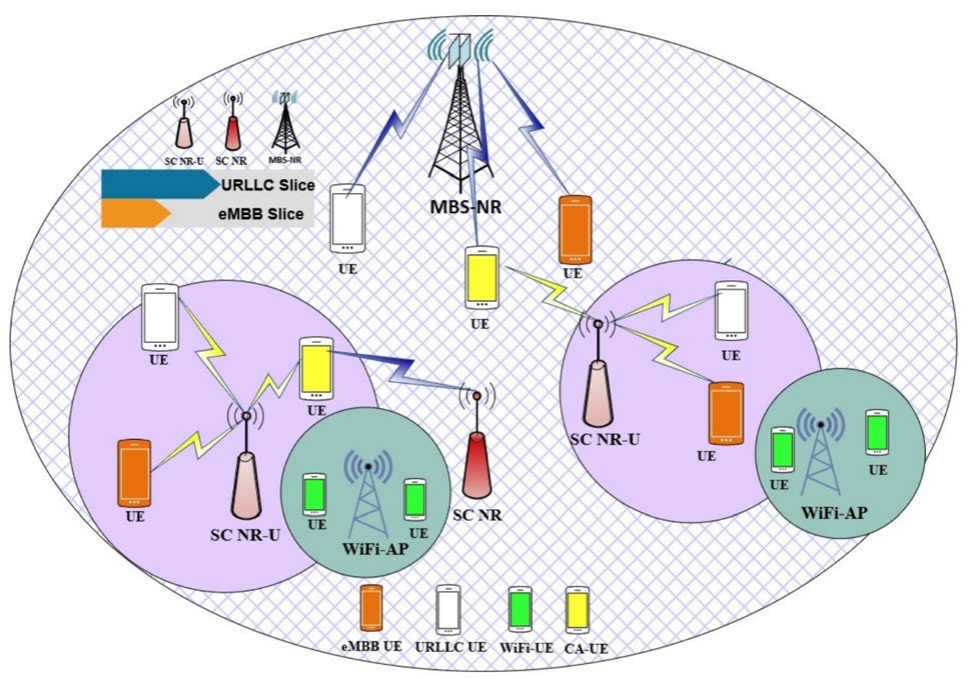
System model of multi-RAT small cells network slicing based.

better throughput, and lower latency. In^24^, the authors proposed a resource allocation mechanism based on carrier aggregation to meet diverse QoS requirements. However, their work mainly focused on reducing packet delay in 5G services to maximize system performance.

From the reviewed studies, it can be concluded that exploiting both 5G NR licensed and NR-U, considering CA transmission mode, within a multi-slice scalable numerology-based approach is key to balancing traffic load and enhancing capacity while ensuring each user’s SLA.

## System design and problem formulation

In this section, a detailed description of the proposed system design and problem formulation is presented.

**Table 1:** Table of notations.

Notation	Description	Notation	Description
$\omega _j$	Scalable numerology	$Delay_{(u,j,k)}^{\textrm{CA}}$	Achieved average delay by user *u* (CA mode)
$\Delta F_j$	Sub-carrier spacing	$S_{(u,j,m)}$	Satisfaction of user *u* in slice *j* associated with BS
$T_{\text {slot}}^{\text {NR/NR-U}}$	Time slot interval in NR or NR-U	$R_j$	User’s service request
$N_{(m,j)}^{\text {RBmax}}$	Maximum number of RBs assigned for a slice	$r_t$	Reward function in time t
$N_{(m,j)}^{\text {RB}}$	Number of RBs assigned for a slice	$V^*(s)$	State value function
$\alpha _j$	Slice resource ratio	$A^*(s,a)$	Action value function
$R_{(u,j,m)}^{\text {NR}}$	Achievable downlink data rate of user *u* from NR-MBS/SC	$R_c$	Cumulative discounted reward (CDR)
$R_{(u,j,m)}^{\text {NR-U}}$	Achievable downlink data rate of user *u* from NR-U SC	$\gamma$	Discount factor (DF)
$Rt_{(u,j,m)}^{\text {phy}}$	Calcualted physical rate	$M^{\text {epi}}$	Number of episodes
$U_j^{\text {NR}}$	Total number of users per slice *j* associated with NR SC	$U_u(s^R)$	User’s utility function
$U_j^{\text {NR-U}}$	Total number of users per slice *j* associated with NR-U SC	$\rho _u^t(x,y)$	Regret of player *u* choosing *y* instead of *x* at time *t*
$U_{\text {wap}}$	Total number of users associated with WiFi-AP	$O_u^t(x,y)$	Payoff for player *u*
$R_{(u,j,m)}^{\text {Mode}}$	Achievable downlink data rate of user *u* (mode-based)	$p_u^{t+1}(y)$	Probability of player *u* choosing action *y* at $t+1$
$Delay_{(u,j,m)}^{\text {NR}}$	Achieved average delay in NR	$\bar{z}_t$	Empirical distribution (ED)
$Delay_{(u,j,m)}^{\text {NR-U}}$	Achieved average delay in NR-U	$\mathscr {C}_{OC}^{\mathrm {NR-U}}$	Channel occupancy in NR-U

### System description

In this paper, we consider a HetNet as shown in Fig. 1 consisting of an MBS overlaid with multiple SCs. Some SCs operate in the licensed NR band, while others operate in the unlicensed NR-U band.

A set that represents these different access points is denoted by$\mathscr {M} = \{0,1,2,\dots ,M\}$, where $m=0$ represents the MBS and $m \in \mathscr {M}$. The next $N_{NR}$ SCs represent the NR-SCs, followed by $N_{NR-U}$ SCs which represent the NR-U SCs. Therefore, the set $\mathscr {M}$ has a cardinality of $M = 1 + N_{NR} + N_{NR-U}$, which represents the total number of physical 6G nodes existing in the network.

WiFi access points (WAPs) are assumed to co-exist on the unlicensed band. To represent WAPs, we define a set $\Psi = \{1,2,\dots ,W\}$, where $w \in \Psi$. Since SCs can operate under both the NR band and the NR-U band, three downlink transmission modes of operation are defined. In Mode 1 (NR mode), the user is served through NR-SCs. In Mode 2 (NR-U mode), the user is served through NR-U SCs. In Mode 3 (CA mode), the user can receive aggregated downlink transmission from both NR-SCs and NR-U SCs. Without loss of generality, we define three subsets to denote each mode of operation. The set of NR-SCs is denoted by $\mathscr {B} = \{1,2,\dots ,N_{NR}\}$, where $\mathscr {B} \subset \mathscr {M}$. The set of NR-U SCs is denoted by $\mathscr {F} = \{N_{NR}+1, N_{NR}+2, \dots , M\}$, with cardinality $|\mathscr {F}| = M - N_{NR}$, where $\mathscr {F} \subset \mathscr {M}$. Moreover, the set that represents CA between NR and NR-U SCs is denoted by $\mathscr {C} = \{N_{NR}, N_{NR}+1\}$.

The NR SCs adopt Orthogonal Frequency Division Multiple Access (OFDMA), while NR-U SCs adopt the Listen-Before-Talk (LBT) scheme, which involves a Clear Channel Assessment (CCA) to determine whether the channel is busy or idle. WiFi adopts the IEEE 802.11 Distributed Coordination Function (DCF), which is based on the Carrier Sense Multiple Access with Collision Avoidance (CSMA/CA) protocol for channel access.

A set of slices $\xi = \{1,2\}$ is introduced to support RAN slicing in the MBS and SCs. Each slice defines a specific service managed by the mobile Service Provider (SP). In addition, a scalable numerology access scheme is adopted, where each slice employs a different numerology based on its requirements. The set of numerologies, which dictates the subcarrier spacing within the associated radio spectrum, is defined as $\mathscr {X}_j = \{0,1,2,3\}$, where $j \in \xi$.

Users are uniformly distributed under the coverage of the MBS and SCs. A set of users is defined as $\mathscr {U} = \{1,2,\dots ,U\}$, where $u \in \mathscr {U}$. The number of users associated with slice $j \in \xi$ in the MBS or NR-SCs is denoted by $U_j^{NR}$, while the number of users associated with slice $j \in \xi$ in NR-U SCs is denoted by $U_j^{NR-U}$. In addition, the number of users associated with WAPs coexisting on the unlicensed band is denoted by $U_{wap}$.

### Problem formulation

Our goal is to fulfill each user service request requirement, ensuring that users requesting URLLC services meet their low-latency requirement, while users seeking eMBB services achieve the desired data rates. To solve this problem, a joint optimization problem is formulated and defined to maximize users’ satisfaction in the network. The joint optimization problem aims to identify an efficient allocation ratio $\alpha _j$ for each slice in each SC within NR and NR-U, determine the optimal scalable numerology value $\omega _j$, where $\omega _j \in \mathscr {X}_j$ and solve the user association problem.

As the service request requirements of each user can be in the form of a minimum delay for URLLC service or high data rate for eMBB services, the satisfaction of user $u$ in slice $j$ associated with BS $m \in \mathscr {M}$ is represented by an increasing concave function, expressed as follows:

1 $$\begin{aligned} S_{u,j,m} = R_j \left( 1 - e^{-\frac{R_{u,j,m}^{Mode}}{R_{\text {ref}}}} \right) + (1 - R_j) \left( 1 - e^{-\frac{D_{\text {ref}}}{D_{u,j,m}^{Mode}}} \right) , \end{aligned}$$

where $R_j$ denotes each user’s service request. $R_j$ is a binary variable, where $R_j = 0$ when user $u$ requests URLLC service, and $R_j = 1$ when user $u$ requests eMBB service. $D_{\text {ref}}$ and $R_{\text {ref}}$ are defined as the minimum average delay and minimum average data rate that a user $u$ can achieve, respectively. In addition, $R_{u,j,m}^{Mode}$ is defined as the achievable downlink data rate based on the transmission mode selected, while $D_{u,j,m}^{Mode}$ is defined as the achievable average delay based on the selected transmission mode. Both will be explained and modeled in the system model section. The satisfaction function in (1) is modeled using exponential saturation functions. Specifically, the first term is monotonically increasing and concave in the achievable data rate, while the second term increases as the delay decreases, reflecting the URLLC requirement. This equation ensures that eMBB users are rewarded by higher data rates and URLLC users by lower latency, guaranteeing their respective QoS demands. Consequently, the user’s satisfaction maximization problem is formulated as follows;

2 $$\begin{aligned} {\textbf {OPT:}} \quad \max _{x,\;\omega _j,\;\alpha _j} \sum _{m \in \mathscr {M}} \sum _{j \in \xi } \sum _{u \in \mathscr {U}} x_{u,j,m} \, S_{u,j,m}, \end{aligned}$$

subject to

2a $$\begin{aligned}&\sum _{m \in \mathscr {M}} x_{u,j,m} = 1, \quad \forall u \in \mathscr {U}, \end{aligned}$$

2b $$\begin{aligned}&\sum _{j \in \xi } x_{u,j,m} = 1, \quad \forall u \in \mathscr {U}, \end{aligned}$$

2c $$\begin{aligned}&x_{u,j,m} = \{0,1\}, \quad \forall u \in \mathscr {U}, \; m \in \mathscr {M}, \end{aligned}$$

2d $$\begin{aligned}&R_{u,j,m}^{\text {Mode}} \ge R_{\text {ref}}, \quad \forall u \in \mathscr {U}, \end{aligned}$$

2e $$\begin{aligned}&Delay_{u,j,m}^{\text {Mode}} \le D_{\text {ref}}, \quad \forall u \in \mathscr {U}. \end{aligned}$$

Where, constraint (2a) expresses that user *u* can associate with only one BS, either an MBS or an SC; while constraint (2b) expresses that user *u* can associate with only one slice in the chosen BS. Without loss of generality, a user cannot associate with two slices per one BS, as each slice represents a different service. Constraint (2c) denotes the association index, where the association index $x_{u,j,m} = 0$ indicates that user *u* is unassociated with BS *m*, while $x_{u,j,m} = 1$ indicates that user *u* is associated with BS *m*.

## System model

In this section, a detailed description of slice ratio resource allocation and scalable numerology is presented. This is followed by a detailed analysis of rate and delay models in both NR and NR-U.

### Slice ratio and scalable numerology formulation

Our system model represents two RAN slices: URLLC slice and eMBB Slice. Generally, each slice represents two different services with two different aims; the URLLC services require low latency combined with high reliability, while eMBB services require high data rates combined with a moderate reliability level. Each slice can be applied on both NR RAT and NR-U RAT.

To accommodate this, our proposed model adopts 5G scalable numerology. The scalable numerology frame structure flexibly supports the heterogeneous services, where each numerology represents different multi-carrier modulation parameters that define different subcarrier spacing and slot duration. Based on this, a scalable numerology set $\mathscr {X}_j = \{0,1,2,3\}$ as shown in Table 2 is adopted. Here, $\omega _j = 0$, $\omega _j \in \mathscr {X}_j$ supports the eMBB slice, while $\omega _j \in \{1,2,3\}$ varies to support URLLC slices. A variable $\omega _j$ results in a variable subcarrier spacing $\Delta F_j$ and slot interval $T_{\text {slot}}^{\text {NR/NR-U}}$, given by^14^:

3 $$\begin{aligned} & \Delta F_j = \Delta F_0 \times 2^{\omega _j}, \end{aligned}$$

4 $$\begin{aligned} & T_{\text {slot}}^{\text {NR/NR-U}} = \frac{T_{\text {slot},0}^{\text {NR/NR-U}}}{2^{\omega _j}}, \end{aligned}$$

where $\Delta F_0 = 15$ kHz and $T_{\text {slot},0}^{\text {NR/NR-U}} = 1$ ms.

To support eMBB services, which require a high data rate, we adopt CA technology to maintain the user’s QoS by allowing the user’s data to be transmitted either on NR (M1 mode), NR-U (M2 mode), or in CA mode (M3 mode). The CA technology exploits the unlicensed band to support the NR network in high-load scenarios. Adopting both scalable numerology and CA technology efficiently balances the diverse users’ service requests.

**Table 2:** NR/NR-U scalable numerology^14^.

$\omega _j$	$\Delta F_j$	$T_{\text {slot}}^{\text {NR/NR-U}}$
0	15 kHz	1 ms
1	30 kHz	0.5 ms
2	60 kHz	0.25 ms
3	120 kHz	0.125 ms

In order to satisfy each user service request, we denote each user’s service request as slice preference. The number of users’ requests *R* preference to a slice in a SC *m* will affect the number of logical RBs in each slice in each SC *m*. Based on this, the maximum number of RBs assigned for each slice *j* from SC *m* can be calculated as follows:

5 $$\begin{aligned} N_{m,j}^{\text {RBmax}} = \frac{B}{\Delta F_j}, \end{aligned}$$

where *B* is the bandwidth of SC *m*.

In addition, the number of RBs assigned for a slice *j* considering other slices’ allocated resources in SC *m* is presented as:

6 $$\begin{aligned} N_{m,j}^{\text {RB}} = \alpha _j N_{m,j}^{\text {RBmax}}, \end{aligned}$$

where $\alpha _j$ is the ratio of RBs allocated to slice *j*, which depends on other slices’ ratios and ranges $0 \le \alpha _j \le 1$. The value of $\alpha _j$ must give $N_{m,j}^{\text {RB}}$ an integer value.

Furthermore, if SC *m* supports two slices and the number of RBs allocated to the first slice can be determined using 6, then the number of RBs assigned to the second slice can be calculated as follows:

7 $$\begin{aligned} N_{m,j', \; j' \ne j}^{\text {RB}} = (1 - \alpha _j) N_{m,j}^{\text {RBmax}}. \end{aligned}$$

## Unlicensed band channel access scheme

In this section, the difference between channel access mechanisms in NR-U and WiFi is studied, highlighting this difference to accommodate WiFi coexistence in our paper and how this will affect our proposed model in terms of contention.

1. **IEEE 802.11 (WiFi) channel access mechanism** IEEE 802.11-based systems employ the Distributed Coordination Function (DCF) mechanism, which is based on Carrier Sense Multiple Access with Collision Avoidance (CSMA/CA), to provide fair access among all WiFi Access Points (APs) sharing the same network. The CSMA/CA process^25^ operates Clear Channel Assessments (CCA) followed by an exponential back-off mechanism to avoid collisions among WiFi APs as follows: the WiFi AP first monitors the channel by checking if it is clear for a Distributed Inter-Frame Space (DIFS) duration. If the channel is free, the WiFi AP transmits data immediately. On the other hand, if the channel is busy, the WiFi AP keeps monitoring the channel until it is idle for a DIFS duration and then initiates the back-off process. The back-off process begins with the choice of a random back-off interval $\beta _{\text {off}} = \text {rand}[0, CW]$, where *CW* is the contention window, initially set to a minimum value $CW_{\min }$. If the channel is sensed idle, the AP decrements the back-off counter for a CCA slot duration; otherwise, if the channel is sensed busy, the counter is paused until the channel is sensed idle again for a DIFS period. The transmission process begins when $\beta _{\text {off}}$ reaches zero, and the initiating WiFi AP can start transmitting for a duration called the Transmit Opportunity (TXOP), which mainly depends on Access Categories (AC)^25^. If retransmission is needed, the contention window *CW* is doubled until it reaches $CW_{\max }$.
2. **NR-U channel access mechanism** For NR-U, channel access relies on the Listen-Before-Talk (LBT) procedure, defined by 3GPP. The process begins with an Initial CCA (ICCA) check, during which the small cell observes the channel for a time interval $T_{\text {idle}}$. If the channel remains idle, the Extended CCA (ECCA) phase is initiated, applying a random backoff process similar to that in Wi-Fi. If transmission fails (meaning no acknowledgment (ACK) is received) the contention window *CW* is doubled up to $CW_{\max }$. Four categories of LBT are defined^26^, ranging from immediate access (Cat 1) to variable backoff with a dynamic contention window (Cat 4). In this work, Cat 4 LBT is considered, where successful access allows transmission only for a bounded interval known as the Channel Occupancy Time (COT), which is mainly based on Channel Access Priority Class (CAPC).

## Rate and delay analysis in NR and NR-U slicing

Our system model, as described earlier, adopts network slicing in both NR and NR-U. Two slices are defined: the URLLC slice, which is characterized by restricted delay requirements, and the eMBB slice, which mainly focuses on achieving high data rates. To ensure that each slice meets its needed requirements, the rate model and the delay model for eMBB and URLLC are studied, respectively, in both NR and NR-U.

### eMBB slice model in NR

In order to achieve the eMBB slice requirement in NR, we need to calculate the average SINR/SNR received by any user *u* from an MBS or NR-SC. For this, the average received SINR by user *u* from an MBS $m \in \mathscr {M}$ and $m=0$ can be expressed as^20^:

8 $$\begin{aligned} \text {SINR}_{(0,u)}^{NR} = \frac{P_{0} G_{(0,u)}}{\sum _{i \in \mathscr {B}} P_i G_{(i,u)} + \sigma ^2}, \; i \in \mathscr {B}, \end{aligned}$$

where $P_{0}$ denotes the transmitted power from the MBS, while $P_i$ denotes the average transmitted power from interfering NR-SC $i \in \mathscr {B}$; $G_{(0,u)}$ and $G_{(i,u)}$ represent the average channel gains between the MBS $m \in \mathscr {M}$ and user *u*, and between the interfering NR-SC $i \in \mathscr {B}$ and user *u*, respectively; while $\sigma ^2$ denotes the additive noise power. Moreover, the average SINR received by user *u* from NR-SC $m \in \mathscr {B}$ can be calculated as follows^20^:

9 $$\begin{aligned} \text {SINR}_{(m,u)}^{NR} = \frac{P_m G_{(m,u)}}{P_0 G_{(0,u)} + \sum _{\begin{array}{c} i \in \mathscr {B} \\ i \ne m \end{array}} \left( P_i G_{(i,u)}\right) + \sigma ^2}, \; i \in \mathscr {B}. \end{aligned}$$

Where, $P_m$ is the transmitted power from NR-SC $m \in \mathscr {B}$; $G_{(m,u)}$ represents the average channel gain between the NR-SC $m \in \mathscr {B}$ and user *u*, while $G_{(i,u)}$ and $G_{(0,u)}$ denote the average channel gains between interfering NR-SC $i \in \mathscr {B}, i \ne m$ and user *u*, and between the interfering MBS $m \in \mathscr {M}$ and user *u*, respectively. Based on the above, the achievable downlink data rate achieved by user *u* from $m \in \mathscr {M}/\mathscr {F}$ in slice *j* is represented as follows^27^:

10 $$\begin{aligned} R_{(u,j,m)}^{NR} = \frac{N_{\text {SUB}}^{sc} \, N_{\text {slot}}^{sc} \, N_{(u,m)}^{bits} \, N_{(m,j)}^{RB} \, CR_{(u,m)}^{NR}}{U_(j,m)^{NR} \, T_{\text {slot}}^{(NR/NR\!-\!U)}}, \; m \in \mathscr {M}/\mathscr {F}. \end{aligned}$$

Where, $N_{\text {SUB}}^{sc}$ represents the number of sub-carriers per one resource block, $N_{\text {slot}}^{sc}$ represents the number of slots per one sub-frame; $N_{(u,m)}^{bits}$ represents the number of bits per symbol, $N_{(m,j)}^{RB}$ is the total number of RBs in slice *j* in BS $m \in \mathscr {M}/\mathscr {F}$; $CR_{(u,m)}^{NR}$ is the coding rate of user *u*. Both $N_{(u,m)}^{bits}$ and $CR_{(u,m)}^{NR}$ can be calculated based on the Channel Quality Indicator (CQI), which is determined by the SINR achieved at user *u*. $U_(j,m)^{NR}$ denotes the total number of users per slice *j* associated to the NR cell $m \in \mathscr {M}/\mathscr {F}$, while $T_{\text {slot}}^{(NR/NR\!-\!U)}$ is the time slot interval and can be determined using 4.

### eMBB slice model in NR-U

Moreover, in NR-U SC, DCF channel contention is considered, hence, interference from other NR-U SCs is neglected. Thus the average SNR received by user *u* from NR-U SC $m \in \mathscr {F}$ can be represented as follows^20^:

11 $$\begin{aligned} \text {SNR}_{m,u}^{(NR\!-\!U)} = \frac{P_m G_{m,u}}{\sigma ^2}, \quad m \in \mathscr {F}. \end{aligned}$$

Where, $P_m$ is the transmitted power from NR-U SC $m \in \mathscr {F}$ and $G_{m,u}$ denotes the average channel gain of NR-U SC $m \in \mathscr {F}$ to user *u*. As we adopted CA in our presented system model, the average SINR received by user *u* from NR SC will be calculated using 9, while the average SNR received by user *u* from NR-U SC is calculated using 11.

Moreover, as NR-U SC adopting Cat4 LBT process, a 2-D Markov chain model is presented with a 2-tuple (z,y) to present the state of procedure^25^ which can be described as follows:

- *z*: is the back-off stage order, defined as $z \in \left( 0, \text {max}_m + \eta _m \right)$, where $\text {max}_m$ is the maximum back-off stage order for $m \in \mathscr {F}$, represented in $CW_{(m,\text {max}_m)}$, and $\eta _m$ is the maximum number of retransmissions.
- *y*: is the back-off counter value, defined as $y \in \left( 0, CW_{(m,z)} - 1\right)$, where $CW_{(m,z)} - 1$ denotes the maximum value of the back-off counter at stage *z*.

From these, the contention window (CW) of NR-U SC at stage *z* is given by^25^:

12 $$\begin{aligned} CW_{(m,z)} = {\left\{ \begin{array}{ll} 2^z \, CW_{(m,0)}, & 0 \le z \le \text {max}_m, \\[6pt] 2^{\text {max}_m} \, CW_{(m,0)}, & \text {max}_m \le z \le \text {max}_m + \eta _m, \end{array}\right. } \end{aligned}$$

Where $CW_{(m,0)}$ represents the minimum contention window at stage *z*. Moreover, the probability that NR-U SC *m* transmits a data packet on the channel is represented as follows^25^:

13 $$\begin{aligned} p_m = \sum _{z=0}^{\text {max}_m + \eta _m} b_{(z,0)} = \frac{1 - \left( p_{(m,c)}\right) ^{\text {max}_m + \eta _m + 1}}{1 - p_{(m,c)}} \, b_{0,0}, \end{aligned}$$

where $b_{(z,y)}$ is defined as the steady-state probability in the two-dimensional Markov chain model ^25^. Thus, $b_{(z,0)}$ represents the probability that the NR-U SC is at back-off stage *z* with back-off counter value zero, while $b_{0,0}$ denotes the initial state probability where both the back-off stage and back-off counter values are zero.

On the other side, the collision probability $p_{(m,c)}$ of a data packet transmitted by an NR-U SC on a channel, when at least one of the remaining $N_{\text {NR-U}} - 1$ NR-U SCs and $N_{\text {WAP}}$ WAPs transmits a data packet in the same time slot, can be defined as follows^25^:

14 $$\begin{aligned} p_{(m,c)} = 1 - \left( 1 - p_m \right) ^{N_{\text {NR-U}} - 1} \left( 1 - p_w \right) ^{N_{\text {WAP}}}, \end{aligned}$$

where $p_w$ is the probability that a WAP transmits a data packet on the channel and can be calculated as in ^25^. In the light of the above, the achievable downlink data rate achieved by user *u* in an NR-U SC, which is quantified as the total number of data bits successfully transmitted by an NR-U SC $m \in \mathscr {F}$ in a given time slot in slice *j* considering the MAC layer effect, can be represented as follows:

15 $$\begin{aligned} R_{(u,j,m)}^{\text {NR-U}} = \frac{p_{(m,s)}. \, D. R t_{(u,j,m)}^{\text {phy}}}{T_{\text {NR-U}}}, \quad \forall m \in \mathscr {F}, \, j \in {\xi }. \end{aligned}$$

Where, $p_{(m,s)}$ is the probability that the NR-U SC data packet is successfully transmitted and can be expressed as follows:

16 $$\begin{aligned} p_{(m,s)} = N_{\mathrm {NR\!-\!U}} \, p_m \, (1 - p_m)^{N_{\mathrm {NR\!-\!U}} - 1} \, (1 - p_w)^{N_{\textrm{WAP}}}, \end{aligned}$$

In addition, *D* is the maximum allowed size of user *u* packet, and $Rt_{(u,j,m)}^{\textrm{phy}}$ represents the NR-U SC physical rate and can be represented as follows:

17 $$\begin{aligned} Rt_{(u,j,m)}^{\textrm{phy}} = \frac{N_{\textrm{SUB}}^{\textrm{sc}} \, N_{\textrm{slot}}^{\textrm{sc}} \, N_{(u,m)}^{\textrm{bits}} \, N_{(m,j)}^{\textrm{RB}} \, CR_{(u,m)}^{\mathrm {NR\!-\!U}}}{U_{(j,m)}^{\mathrm {NR\!-\!U}} \, T_{\textrm{slot}}^{\mathrm {NR\backslash NR\!-\!U}}}, \quad \forall m \in \mathscr {F}, \; j \in \xi , \; \end{aligned}$$

Where, $N_{\textrm{SUB}}^{\textrm{sc}}$ represents the number of sub-carriers per one resource block, $N_{\textrm{slot}}^{\textrm{sc}}$ represents the number of slots per one sub-frame; $N_{(u,m)}^{\textrm{bits}}$ represents the number of bits per symbol, $N_{(m,j)}^{\textrm{RB}}$ is the total number of RB in slice *j* in BS $m \in \mathscr {F}$; $CR_{(u,m)}^{\mathrm {NR\!-\!U}}$ is the coding rate of user *u*. $N_{(u,m)}^{\textrm{bits}}$ and $CR_{(u,m)}^{\mathrm {NR\!-\!U}}$ can both be calculated based on the CQI, which is determined by the SNR achieved at user *u*. $U_{(j,m)}^{\mathrm {NR\!-\!U}}$ is the total number of users per slice *j* associated with NR-U SC $m \in \mathscr {F}$, while $T_{\textrm{slot}}^{\mathrm {NR\backslash NR\!-\!U}}$ is the time slot interval and can be determined using 4. In addition, $T_{\text {NR-U}}$ is the time interval between two successive slots and can be calculated as follows^25^:

18 $$\begin{aligned} T_{\text {NR-U}} = \delta p_{\text {idle}} + p_{(m,s)} \big [ T_{\text {mcot}} + T_{\text {DIFS}} + \delta \big ] + p_{(w,s)} T_{ws} + p_{(m,c)} \big [ T_{\text {mcot}} + T_{\text {DIFS}} + \delta \big ] + p_{(w,c)} T_{wc} + p_{(m,w,c)} T_{(m,w,c)}, \end{aligned}$$

where $\delta$ is the length of one time slot, $p_{\text {idle}}$ is the probability that the channel is sensed to be idle and can be calculated as follows^25^:

19 $$\begin{aligned} p_{\text {idle}} = (1 - p_m)^{N_{\text {NR-U}}} (1 - p_w)^{N_{\text {WAP}}}, \end{aligned}$$

Moreover, $T_{\text {mcot}}$ is the maximum channel occupancy time of NR-U SC, $T_{\text {DIFS}}$ denotes the DCF Inter-Frame Space (DIFS) short frame, while $p_{(w,s)}$ is the probability that a WAP transmits successfully on the channel and can be represented as follows^25^:

20 $$\begin{aligned} p_{(w,s)} = N_{\text {WAP}} \, p_w \, (1 - p_w)^{N_{\text {WAP}} - 1} (1 - p_m)^{N_{\text {NR-U}}}, \end{aligned}$$

In addition, $T_{ws}$ denotes the channel occupation time by a WAP and can be represented as follows^27^:

21 $$\begin{aligned} T_{ws} = T_{\text {RTS}} + T_{\text {CTS}} + T_{\text {ACK}} + 3T_{\text {SIFS}} + T_{\text {DIFS}} + \delta + T_H + T_D, \end{aligned}$$

Where $T_{\text {RTS}}$ is the duration of the Request to Send (RTS) short frame, and $T_{\text {CTS}}$, $T_{\text {ACK}}$, and $T_{\text {SIFS}}$ are the durations of the Clear to Send (CTS) short frame, the Acknowledgment (ACK) short frame, and the Short Inter-Frame Space (SIFS), respectively. Meanwhile, $T_H$ and $T_D$ denote the WiFi packet header transmission time and the WiFi packet payload transmission time, respectively.

In addition, $p_{(w,c)}$ denotes the probability of collision between WAPs and can be represented as follows^27^:

22 $$\begin{aligned} p_{(w,c)} = (1 - p_m)^{N_{\text {NR-U}}} \Big [ 1 - (1 - p_w)^{N_{\text {WAP}}} - N_{\text {WAP}} p_w (1 - p_w)^{N_{\text {WAP}} - 1} \Big ], \end{aligned}$$

While $T_{wc}$ is the channel occupation time in case of collision, and can be represented as follows^25^:

23 $$\begin{aligned} T_{wc} = T_{\text {RTS}} + T_{\text {DIFS}} + \delta , \end{aligned}$$

While $p_{(m,w,c)}$ represents the probability of collision between one NR-U SC and one WAP contending on the channel simultaneously, and can be represented as follows^25^:

24 $$\begin{aligned} p_{(m,w,c)} = 1 - p_{\text {idle}} - p_{(w,c)} - p_{(m,c)} - p_{(m,s)} - p_{(w,s)}, \end{aligned}$$

Consequently, $T_{(m,w,c)}$ is the occupation time in which the channel is occupied by a collision between a NR-U SC and a WAP and can be given as^25^:

25 $$\begin{aligned} T_{(m,w,c)} = \max \big ( T_{wc}, T_{mc} \big ). \end{aligned}$$

### eMBB slice CA model between NR and NR-U

As we adopted CA in our presented system model, if user *u* is associated to an SC using CA (M3 mode), the achievable data rate of user *u* will be expressed as the sum of the achieved date rate from NR SC and NR-U SC as follows:

26 $$\begin{aligned} R_{(u,j,m)}^{\text {Mode}} = \gamma R_{(u,j,m)}^{\text {NR}} + \beta R_{(u,j,m)}^{\text {NR-U}}, \quad \forall u \in U_j, \; m \in \mathscr {M}, \; j \in \xi , \end{aligned}$$

where $\gamma$ and $\beta$ are binary variables that represent the user’s association, reflecting the transmission mode selection. They are defined as:

27 $$\begin{aligned} (\gamma , \beta ) = {\left\{ \begin{array}{ll} (1,0), & \text {if SC }m\text { operates in M1, } m \in \mathscr {B}, \\ (0,1), & \text {if SC }m\text { operates in M2, } m \in \mathscr {F}, \\ (1,1), & \text {if SC }m\text { operates in M3, } m \in \mathscr {C}, \end{array}\right. } \end{aligned}$$

### URLLC slice model in NR

The average delay to transmit a packet, which is defined as the number of transmission time intervals, can be calculated as follows:

28 $$\begin{aligned} \text {Delay}_{(u,j,m)}^{\text {NR}} = \Bigg \lceil \frac{\text {Packet}_{\text {size}}}{N_{\text {SUB}}^{\text {sc}} \, N_{\text {slot}}^{\text {sc}} \times N_{(m,j)}^{\text {RB}} \times \psi ^{\text {NR}}} \times U_{(j,m)}^{\text {NR}} \Bigg \rceil T_{\textrm{slot}}^{\mathrm {NR\backslash NR\!-\!U}}, \quad m \in \mathscr {M} /\mathscr {F}, \; j \in \xi , \end{aligned}$$

where $\psi ^{\text {NR}}$ is defined as $\psi ^{\text {NR}} = N_{(u,m)}^{\text {bits}} \times CR_{(u,m)}^{\text {NR}}$.

### URLLC slice model in NR-U

The average delay to successfully transmit a packet in NR-U is defined as the time taken once an NR-U SC begins to contend for the channel until a data packet is transmitted successfully, and can be expressed as follows:

29 $$\begin{aligned} \text {Delay}_{(u,j,m)}^{\text {NR-U}} = \left\lceil \llceil \frac{\text {Packet}_{\text {size}}}{N_{\text {SUB}}^{\text {sc}} \, N_{\text {slot}}^{\text {sc}} \times N_{(m,j)}^{\text {RB}} \times \psi ^{\text {NR-U}} \times \mathscr {C}_{OC}^{\text {NR-U}} } \times U_{(j,m)}^{\text {NR-U}} \right\rceil \rrceil T_{\textrm{slot}}^{\mathrm {NR\backslash NR\!-\!U}}, \quad m \in \mathscr {F}, \; j \in \xi , \end{aligned}$$

where $\psi ^{\text {NR-U}}$ is defined as: $\psi ^{\text {NR-U}} = N_{(u,m)}^{\text {bits}} \times CR_{(u,m)}^{\text {NR-U}}$. The channel occupancy in NR-U, $\mathscr {C}_{OC}^{\text {NR-U}}$, can be represented as follows:

30 $$\begin{aligned} \mathscr {C}_{OC}^{\text {NR-U}} = \frac{R_{(u,j,m)}^{\text {NR-U}}}{Rt_{(u,j,m)}^{\text {phy}}}, \end{aligned}$$

### URLLC slice CA model between NR and NR-U

Since we adopted CA in our presented system model, if user *u* is associated to an SC using CA (M3 mode), the aggregated average time delay of user *u* when associated to SC (M3) will be expressed as the maximum time delay of the two total times taken on both NR SC and NR-U SC as follows:

31 $$\begin{aligned} \text {Delay}_{(u,j,k)}^{\text {CA}} = \max \left( T_{I_{\max }}^{\text {delay}}, \; T_{I_{\min }}^{\text {delay}} \right) , \quad k \in \mathscr {C}, \end{aligned}$$

where $\mathscr {C}$ denotes the CA set with index $k \in \mathscr {C}$ and let $T_k$ denote the chosen time slot in the NR/NR-U SC. When $k=1$, it represents $T_{\text {slot}}^{\text {NR}}$, while when $k=2$, it represents $T_{\text {slot}}^{\text {NR-U}}$. Thus, we define the index that corresponds to the minimum time slot value as:

32 $$\begin{aligned} I_{\min } = \arg \min _{k \in \mathscr {C}} \; T_k, \end{aligned}$$

and similarly, $I_{\max }$ defines the index that corresponds to the maximum time slot value as follows:

33 $$\begin{aligned} I_{\max } = \arg \max _{k \in \mathscr {C}} \; T_k. \end{aligned}$$

Based on the previously defined minimum $I_{\min }$ and maximum $I_{\max }$ index values, we can derive a ratio that represents the equivalence time split between the two channels, NR and NR-U. This ratio is defined as:

34 $$\begin{aligned} R_{\text {split}} = \frac{T_{I_{\max }}}{T_{I_{\min }}}, \end{aligned}$$

Consequently, $T_{I_{\min }}^{\text {delay}}$ is represented as follows:

35 $$\begin{aligned} \begin{aligned} T_{I_{\min }}^{\text {delay}} = \Bigg [&\left( \frac{\text {Packet}_{\text {size}}}{R_{\text {split}} \, b_{I_{\min }} + b_{I_{\max }}} \right) ^{-} \cdot R_{\text {split}} \\&+ \min \Bigg ( R_{\text {split}}, \; \left( \frac{\text {mod} \left( \frac{\text {Packet}_{\text {size}}}{R_{\text {split}} \, b_{I_{\min }} + b_{I_{\max }}} \right) }{b_{I_{\min }}} \right) ^{+} \Bigg ) \Bigg ] \; T_{I_{\min }}, \end{aligned} \end{aligned}$$

Where $b_{I_{\max }/I_{\min }}$ represents the number of bits that can be sent in one time slot considering the CQI that can be determined by the SNR achieved at user *u*, and can be defined as follows:

36 $$\begin{aligned} b_{I_{\max }/I_{\min }} = \frac{N_{\text {SUB}}^{\text {sc}} \, N_{\text {slot}}^{\text {sc}} \, N_{(u,k)}^{\text {bits}} \, N_{(k,j)}^{\text {RB}} \, CR_{(u,k)}^{\text {NR-U}}}{U_{(j,m)}^{\text {NR-U}}}, \end{aligned}$$

The term $\left( \frac{\text {Packet}_{\text {size}}}{R_{\text {split}} \, b_{I_{\min }} + b_{I_{\max }}}\right) ^{-}$ represents the total number of simultaneous bits split occurring in the CA mode, where the simultaneous bits split are distributed across the CA channels in equivalent time $T_{I_{\max }}$. Meanwhile, the term $\text {mod}\left( \frac{\text {Packet}_{\text {size}}}{R_{\text {split}} \, b_{I_{\min }} + b_{I_{\max }}}\right)$ defines the remainder number of time slots to be sent on the $I_{\min }$ channel.

On the other side, $T_{I_{\max }}^{\text {delay}}$ is represented as follows:

37 $$\begin{aligned} T_{I_{\max }}^{\text {delay}} = \Bigg [ \left( \frac{\text {Packet}_{\text {size}}}{R_{\text {split}} \, b_{I_{\min }} + b_{I_{\max }}} \right) ^{-} + \left( \frac{\text {Packet}_{\text {size}} \; \text {mod} \; (R_{\text {split}} \, b_{I_{\min }} + b_{I_{\max }})}{R_{\text {split}} \, b_{I_{\min }}} \right) ^{-} \Bigg ] \; T_{I_{\max }}, \end{aligned}$$

where the term $\left( \frac{\text {Packet}_{\text {size}} \; \text {mod} \; (R_{\text {split}} \, b_{I_{\min }} + b_{I_{\max }})}{R_{\text {split}} \, b_{I_{\min }}}\right) ^{-}$ represents whether the last time slot of the channel that has the largest time slot value will exist or not. It has a value of 0 or 1, since the channel with the smallest time slot value is filled first with bits until the number of time slots reaches $R_{\text {split}}$.

based on the above, the achievable average delay based on the selected transmission mode is expressed based on the selected SC as follows:

38 $$\begin{aligned} \text {Delay}_{(u,j,m)}^{\text {Mode}}= {\left\{ \begin{array}{ll} {Delay}_{(u,j,m)}^{\text {NR}}, & \text {if SC }m\text { operates in M1, } m \in \mathscr {B}, \\ {Delay}_{(u,j,m)}^{\text {NR-U}}, & \text {if SC }m\text { operates in M2, } m \in \mathscr {F}, \\ {Delay}_{(u,j,K)}^{\text {CA}}, & \text {if SC }m\text { operates in M3, } k \in \mathscr {C}, \end{array}\right. } \end{aligned}$$

## Proposed model framework

This section introduces a structured iterative framework designed to solve the optimization problem, aiming to maximize user satisfaction. The framework integrates DRL and regret learning techniques to jointly address slice resource allocation, scalable numerology selection, and user association.

First, an initial association process is developed to capture the number of user requests for each slice at each base station (MBS or SC). Each user forms a preference list of base stations whether it falls under NR or NR-U technologies, according to the channel conditions and the type of service required (eMBB or URLLC).

Next, a dynamic resource allocation scheme is applied. Based on the resulting user preferences, the number of users in each slice in each base station is determined and used as input to the DRL algorithm. The DRL dynamically allocates resources at each slice and efficiently chooses the scalable numerology value. The users’ satisfaction is evaluated iteratively; if there is an enhancement, the slice resource ratio and scalable numerology value are updated accordingly. Otherwise, the loop exits, and the resulting slice ratio and scalable numerology values are fed into the proposed regret learning algorithm.

Finally, a regret-based matching algorithm is applied, considering both the resulting slice ratio and scalable numerology values from DDQN algorithm. In a Multi-RAT environment, the problem extends beyond associating users with the most suitable base station (BS) that guarantees user satisfaction-it also involves selecting the appropriate BS with the proper transmission technology, referred to in our model as modes (NR Mode, NR-U Mode, and CA Mode). Therefore, an effective association technique, such as the Regret Learning Algorithm, is essential to jointly select both the optimal transmission mode and the corresponding BS for association. In RLA, users take actions guided by a predefined utility function defined in our proposed model as user’s satisfaction. According to this, the users are re-associated using RLA with the base station that will guarantee satisfaction of each user’s. After this, the users’ satisfaction is checked again; if there is any improvement, the updated associated results are fed to the DDQN algorithm, and the loop continues. Otherwise, if no improvement is achieved, the DRL slice ratio and scalable numerology values are neglected, the regret learning results are ignored, and the loop terminates. The last user association scheme with the slice ratio and scalable numerology values are taken as the final optimized solution.

### Deep reinforcement learning: dueling deep Q-network

In order to efficiently evaluate each slice resource ratio $\alpha _j$ in each SC while jointly determining the optimum scalable numerology $\omega _j$, aiming to satisfy each user *u* service request requirement, a Dueling Deep Q-Learning Network (DDQN) resource slicing algorithm is proposed.

Our proposed DDQN algorithm is designed as a finite Markov Decision Process (MDP), which divides the output layer into two functions: the value function and the advantage function network. The linear combination of these two functions provides the final output of the Q-Network, which is referred to as the MDP quaternion ^28^ and is defined as $(S, A, R, S', \gamma )$, where *S* denotes the discrete set of environmental states, *A* represents the set of all feasible action for an agent (including the current action *a* and the next action $a'$), *R* represents the agent’s reward function, After an action $a_t \in A$ is taken at state $s_t \in S$, a reward $r_t \sim R(\cdot \,|\, s_t, a_t)$ is obtained, $S'$ refers to the next state, and $\gamma \in [0,1]$ is the discount factor.

Accordingly, the discrete set of states in our proposed DDQN algorithm is characterized by two variables: the logical RB ratio $\alpha _j$ assigned to a slice *j* in a SC *m* (where $j=1$ corresponds to the eMBB slice and $j=2$ corresponds to the URLLC slice), and the scalable numerology value $\omega _j$. These two parameters are essential to guarantee that each user’s service requirements are satisfied. Based on this, the value of $\alpha _j$ for one slice allows the calculation of $\alpha _{j', j' \ne j}$ for the other slice using the following:

39 $$\begin{aligned} \alpha _{j', j' \ne j} = \left\lfloor \frac{B - \alpha _j \Delta F_0}{\Delta F_j} \right\rfloor , \end{aligned}$$

Based on the defined state set, the action space is represented as $A = \{ (\alpha _j \pm 1, \; 1 \le \alpha _j \le 100, \; \omega _j \pm 1, \; 0 \le \omega _j \le 3) \}$. When the agent selects an action, the system transits to the next state $S^{\prime }$ with a certain transition probability. The transition probability $\varrho$ is defined as^28^:

40 $$\begin{aligned} \varrho = \left\{ \varrho ^{a}_{s,s^{\prime }} \; \big | \; s, s^{\prime } \in S, \; a \in A \right\} . \end{aligned}$$

Additionally, the reward $r_t \in R$ in the DDQN-learning algorithm is defined as the sum of the satisfaction of all users associated with SCs $m \mathscr {M}$, based on each user’s service preference, as follows:

41 $$\begin{aligned} r_t = \sum _{u,j,m} S_{(u,j,m)}, \; u \in U, \end{aligned}$$

where $S_{(u,j,m)}$ is the user’s satisfaction and can be obtained using (1).

In the presented DDQN-learning algorithm, the agent learns the optimal strategy $\pi ^*$, which corresponds to the state value function $V^*(s)$ and the action value function $A^*(s,a)$, expressed as^28^:

42 $$\begin{aligned} Q^{(\pi ^*)}(s,a) = V^{(\pi ^*)}(s) + A^{(\pi ^*)}(s,a). \end{aligned}$$

After a number of iterations, the target *Q*-value is obtained using the Bellman equation as follows^28^:

43 $$\begin{aligned} Q_t = r + \gamma \max _{a^*} \hat{Q}(s^{\prime }, a^{\prime }, \Theta , \phi , \varphi ), \end{aligned}$$

where $0 \le \gamma \le 1$, $\Theta$ is the shared parameter, $\phi$ is the dominant function parameter, and $\varphi$ is the action-value function parameter. Furthermore, the loss function $L(\Theta )$ is defined as^28^:

44 $$\begin{aligned} L(\Theta ) = \mathbb {E}\Big [ \big (Q_t - Q(s,a,\Theta ,\phi ,\varphi )\big )^2 \Big ]. \end{aligned}$$

where $\mathbb {E}[\cdot ]$ is the expectation with respect to the reward distribution. The formula of the *Q*-value update is represented as follows^28^:

45 $$\begin{aligned} Q(s,a,\Theta ,\phi ,\varphi ) = Q(s,a,\Theta ,\phi ,\varphi ) + \eta \big [ Q_t - Q(s,a,\Theta ,\phi ,\varphi ) \big ], \end{aligned}$$

where $\eta$ is the learning rate. Over time, the agent learns the optimal policy to maximize the discounted rewards. This cumulative discounted reward can be expressed as follows^28^:

46 $$\begin{aligned} R_c = \mathbb {E} \left[ \sum _{i=0}^{\infty } \gamma ^i \, r_{t+i} \right] , \end{aligned}$$

Furthermore, the details of the proposed resource slicing Q-learning algorithm are summarized in Algorithm 1. In Line 1, the initialization process is performed in each episode, given $M^{\text {epi}}$ number of episodes. In Lines 2–3, the number of resource blocks $N_{m,j}^{RB}$ per slice is calculated, which depends on the values of $\alpha _j$ and $\omega _j$. These values can either be initialized or obtained from the previous DDQN-learning algorithm iteration. Finally, in Lines 6–8, the reward $r_t$ is calculated according to (40), given the resulting state $s_{t+1}$, and the state-action table is updated. The algorithm is terminated when the Q-table converges or when a predefined number of iterations is reached.

**Algorithm 1:**
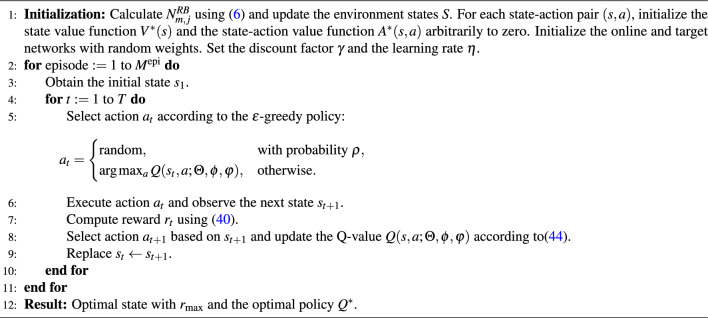
DDQN-Learning based resource-slicing allocation.

### Regret learning algorithm: user-re-association

A regret matching learning approach is developed, named as the *re-association phase*, which addresses the user association problem after determining the optimal slice resource allocation ratio $\alpha _j$ and scalable numerology $\omega _j$ values. The regret learning algorithm ensures convergence toward a generalized equilibrium solution that offers an optimal solution, which extends the classical Nash equilibrium^29^ by allowing the actions of players (i.e., users and BSs) to be correlated. This correlation can improve payoffs and stabilize the system in dynamic environments.

Formally, the user association problem is modeled as a repeated game^29^:

47 $$\begin{aligned} g = \left( U', S_u^R, U_u \right) , \end{aligned}$$

where $U'$ denotes the set of players (the users), $S_u^R \in S^R$ is the set of strategies available to user *u* ,corresponding to the available BSs (MBS and SCS), $S^R$ is the set of joint strategies of all players, and $U_u: S^R \rightarrow \mathbb {R}$ represents the payoff function of player *u* given a joint action $s^R \in S^R$. The utility of user *u* associating with BS *m* is expressed as follows, representing the user’s level of satisfaction expressed in terms of achievable data rate or achievable delay:

48 $$\begin{aligned} U_u(s^R) = S_{u,j,m}, \end{aligned}$$

where $S_{u,j,m}$ can be calculated as in (1). Considering randomized actions according to a Probability Mass Function (PMF) $n \in \mathscr {N}$, the expected payoff of user *u* is given by

49 $$\begin{aligned} U_u(n) = \sum _{s^R \in S^R} n(s^R) \cdot U_u(s^R). \end{aligned}$$

A probability distribution $\pi ^R$ defined over the set of joint strategies $S^R$ is said to form a *correlated equilibrium* if, for every player $u \in U'$ and for every pair of strategies $x,y \in S_u^R$, the following condition expressed as^30^:

50 $$\begin{aligned} \sum _{s^R \in S^R : u = x} \pi ^R(s^R) \, \Big ( U_u(y, s_{-u}^R) - U_u(x, s_{-u}^R) \Big ) \le 0. \end{aligned}$$

This ensures that when strategy *x* is recommended, deviating to another strategy $y \ne x$ yields no positive gain. The core goal of no-regret matching algorithms is to ensure that player u experiences no regret, meaning the probability of selecting a particular strategy is proportional to the regret of not having chosen alternative strategies. To implement this condition, the regret of user *u* for choosing *x* instead of *y* at time *t* is defined as^30^:

51 $$\begin{aligned} \rho _u^t(x,y) \triangleq \max \big ( O_u^t(x,y), 0 \big ), \end{aligned}$$

where $O_u^t(x,y)$ represents the payoff difference had user *u* consistently played *y* instead of *x* in the past^29^:

52 $$\begin{aligned} O_u^t(x,y) = \frac{1}{t} \sum _{T \le t} \Big ( U_u^T(y, s_{-u}^R) - U_u^T(x, s_{-u}^R) \Big ). \end{aligned}$$

Based on these regrets, the probability distribution with which user *u* selects an action at iteration $t+1$ is given by^29^

53 $$\begin{aligned} p_u^{(t+1)}(y) = {\left\{ \begin{array}{ll} \frac{1}{k} \, \rho _u^t(x,y), & y \ne x, \\[6pt] 1 - \sum \limits _{y \in S_u^R, y \ne x} p_u^{(t+1)}(y), & y = x, \end{array}\right. } \end{aligned}$$

where $k > 2MG$ is a constant ensuring positive probabilities, and *G* is the upper bound on $|U(s^R)|$ for all $s^R \in S^R$. At $t=1$, the probability distribution is initialized uniformly over all available strategies.

Over time, the empirical distribution of joint actions up to iteration *t* converges toward a correlated equilibrium, and it is expressed as^30^

54 $$\begin{aligned} \bar{x}_t(s) = \frac{1}{t} N(t, s^R), \end{aligned}$$

where $N(t, s^R)$ denotes the number of times the joint action $s^R$ was selected in the first *t* iterations. Through this iterative regret-matching steps, users usually adapt their association strategies until there is no regret, and the overall system converges to the correlated equilibrium $S^{R*}$.

**Algorithm 2:**
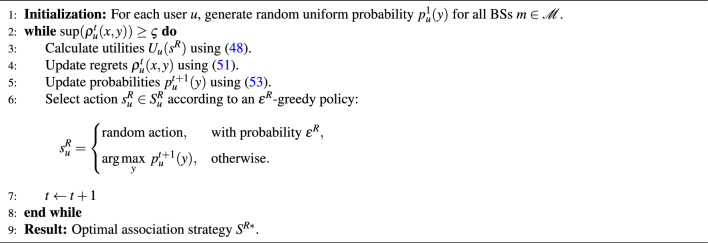
Regret-matching algorithm for user re-association phase.

Accordingly, Algorithm 2 proposes the regret-matching algorithm process for the user re-association phase to find the optimal strategy $S^{R*}$. Starting with the initialization process (Lines 1–4), the utility $U_u(s^R)$ is calculated for each user *u*, then the payoff $O_u^t(x,y)$ is evaluated to update the regrets $\rho _u^t(x,y)$. In Lines 5–6, the probabilities $p_u^{t+1}(y)$ for each user *u* are updated, where the strategy $s_u^R$ is chosen based on the $\epsilon ^R$-greedy policy. The algorithm is repeated until $\rho _u^t(x,y) < \varsigma$, where $\varsigma$ should be properly chosen as in^31^.

## Performance evaluation

This section presents the simulation results used to assess the performance of the proposed framework. We start by describing the simulation setup, mentioning the simulation parameters and configuration details. Then we present our proposed model results.

### Simulation setup

In order to evaluate the proposed framework, the following simulation setup was adopted, where a Multi-RAT HetNet is considered with one MBS and three SCs. One SC deploys NR technology only, one SC deploys NR-U technology, and one SC works on both NR and NR-U technology, which allows three access options that can be denoted by three transmission modes of operation: *M*1 (NR mode), *M*2 (NR-U mode), and *M*3 (NR and NR-U aggregation (CA mode)). The physical SC, which works on both NR and NR-U, can represent three modes of operation, including the aggregate mode (*M*3).

**Table 3:** Simulation parameters.

Parameter	Value	Parameter	Value
NR parameters			
Transmit power of MBS	46 dBm	Transmit power of NR SC	20 dBm
Path loss (MBS–User)	$128.1 + 37.6\log _{10}(d\, [\textrm{km}])$	Path loss (SC–User)	$140.7 + 36.7\log _{10}(d)$
NR bandwidth	20 MHz	Channel bandwidth	180 kHz
Transmission frequency	2 GHz	Noise power	$-174$ dBm/Hz
WLAN parameters			
WLAN bandwidth	20 MHz	Tx power of WiFi-AP	200 mW
Spatial streams	4	Initial contention window (CW)	16
WiFi back-off stages	6	Max. retransmissions	9
PHY header	224 bits	Slot time	9 $\mu$s
NR-U parameters			
NR-U bandwidth	20 MHz	Tx power of NR-U SC	20 dBm
Defer period	34 $\mu$s	Initial contention window (CW)	16
NR-U back-off stages	6	Max. retransmissions	9
Slot time	9 $\mu$s		
Common WLAN and NR-U parameters			
DIFS	34 $\mu$s	SIFS	16 $\mu$s
ACK	112 bits + PHY header	RTS	160 bits + PHY header
CTS	112 bits + PHY header		
Network parameters			
Number of slices	eMBB, URLLC	Packet size	1500 bytes
Numerology (eMBB)	$\mu _j = 0$	Numerology (URLLC)	$\mu _j \in \{1,2,3,4\}$
RB ratio range	$0 \le \alpha _j \le 1$		
DDQN parameters			
$M^{\text {epi}}$	5000	Discount factor $\gamma$	0.9
Min. rate demand $R_{\text {ref}}$	1 Mbps	Min. delay demand $D_{\text {ref}}$	1 ms
Learning rate $\eta$	$2 \times 10^{-8}$		

**Fig. 2:**
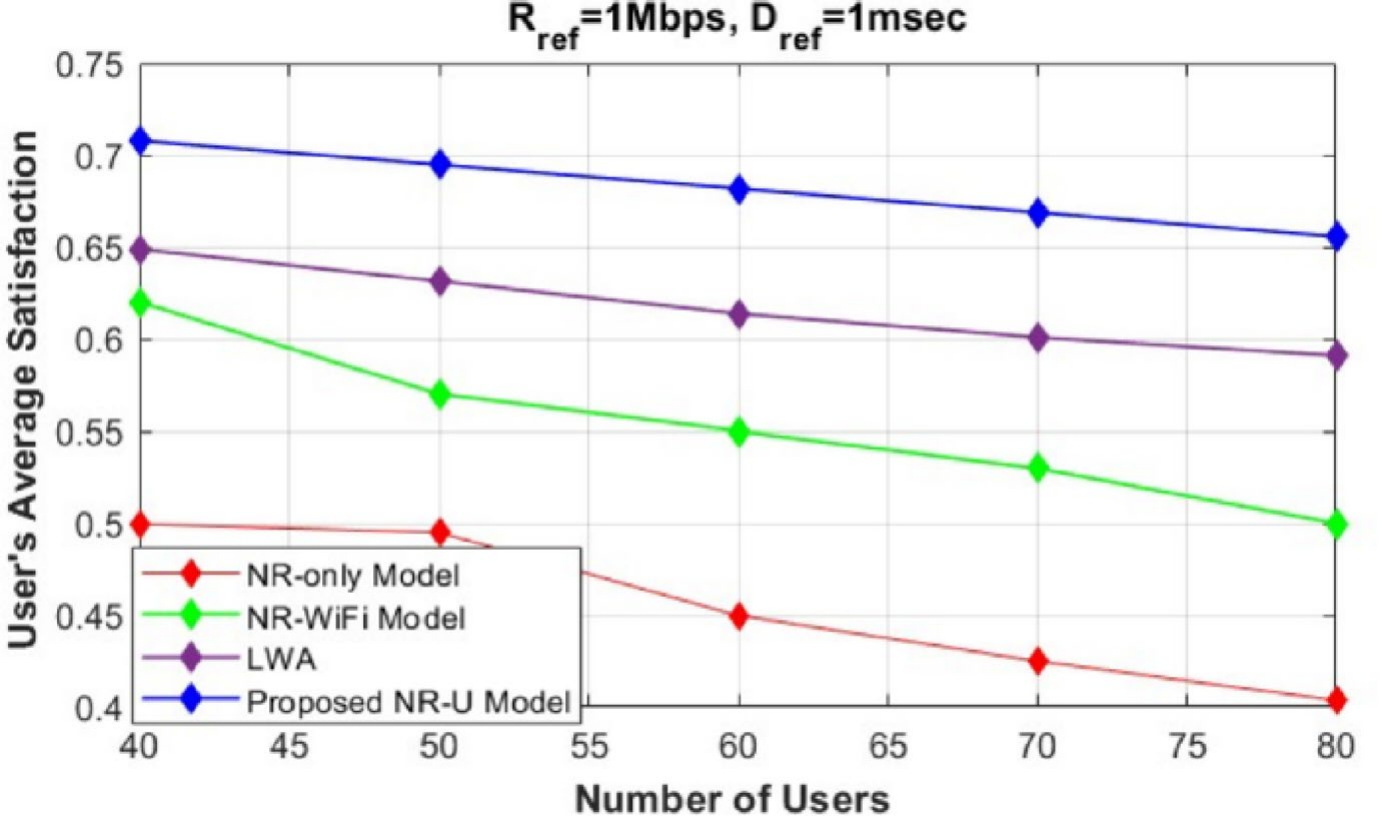
Evaluation of users’ average satisfaction between our proposed model and different baseline approaches (NR-only^32^, NR-WiFi and LWA^20^).

**Fig. 3:**
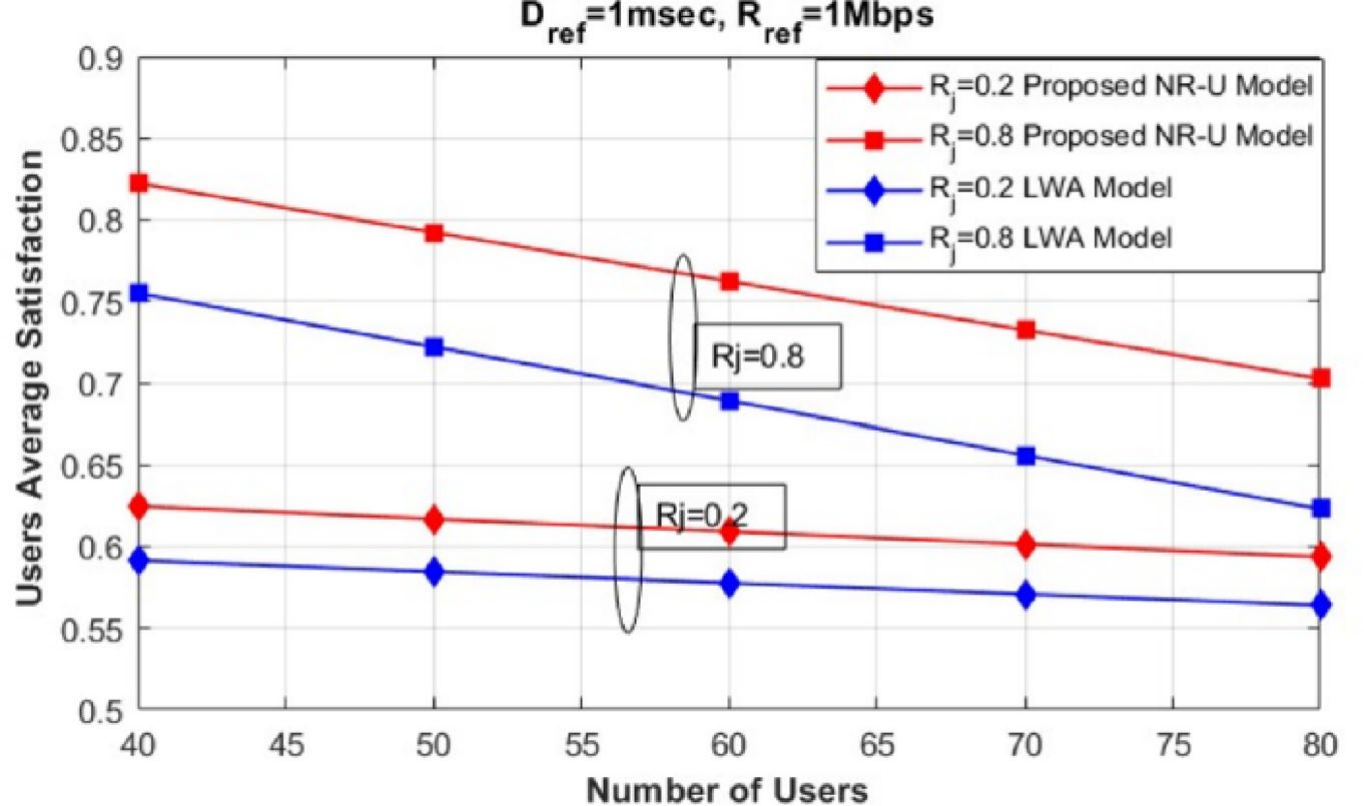
Evaluation of users’ average satisfaction of our proposed model compared to LWA^20^ model in different Users’ service requests ratios.

Furthermore, the two SCs operating on the unlicensed band (*M*2 and NR-U in *M*3) utilize two non-overlapping channels. In addition, a number of WAPs are deployed, each serving multiple associated users $U_{\text {wap}}$, to model coexistence scenarios on the same NR-U non-overlapping channels. This setup reflects realistic spectrum sharing conditions in the unlicensed spectrum. Each SC has a radius of $50\,\text {m}$ and is deployed in an indoor hotspot area of $350 \times 225\,\text {m}^2$ under the coverage of the MBS, which has a radius of $1000\,\text {m}$. A number of users are uniformly distributed under the coverage of the MBS and inside the indoor hotspot area. In addition, each SC is divided into two slices, namely eMBB and URLLC. Moreover, the path loss model for NR-U technology is presented as follows ^33^:

55a $$\begin{aligned} P_{\text {LOS}}&= 32.4 + 20 \log _{10}(f_c) + 17.3 \log _{10}(d), \end{aligned}$$

55b $$\begin{aligned} P_{\text {NLOS}}&= 17.3 + 24.9 \log _{10}(f_c) + 38.3 \log _{10}(d). \end{aligned}$$

In the proposed scenario, we deploy Multi-RAT BSs, comprising an MBS and SCs. This deployment strategy aims to leverage multiple radio access technologies to simultaneously support both eMBB and URLLC users. The objective is to strike an effective balance between delivering high data rates for eMBB users and ensuring low latency for URLLC users.

To provide a fair comparison, our proposed framework is evaluated against three existing models that are widely adopted in the literature. The first is the **NR-only model** ^32^, in which all SCs operate on NR technology. The purpose of comparing our proposed model with this baseline model is to highlight the added value of exploiting the unlicensed band in terms of network capacity. The second model is the **NR–WiFi model**, where half of the SCs operate on NR technology while the other half operate on the unlicensed band using WiFi, disregarding the aggregation transmission mode. Although this approach overcomes the limited NR capacity by leveraging the unlicensed band capacity, it also decreases the network deployment cost; however, it lacks the aggregation transmission mode, which is needed to sustain user demands under highly congested scenarios. The third model considered is the **LWA model** ^20^, where half of the SCs operate on NR technology and the others operate on WiFi, enabling aggregation transmission mode. Compared to the NR–WiFi model, LWA provides better capacity gains, especially in high-demand scenarios, due to its ability to aggregate licensed and unlicensed spectrum. however, LWA does not support network slicing and multi-numerology in the unlicensed band (WiFi), which limits its ability to guarantee each user’s SLA.

For above three models, we ensure the same number of SCs that will be equivalent to SCs in our proposed framework. In addition, to evaluate our proposed framework to other previous models fairly, we simulate our proposed framework without WiFi co-existence users to ensure fair simulation architecture and parameters. While the rest of our simulation results figures consider WiFi co-existence users.

Furthermore, we compared our proposed model using the DDQN algorithm to the Q–learning algorithm and the Deep Q-Network (DQN) algorithm. In addition, our proposed framework is compared to Heuristic-Genetic Algorithm (GA), where the DDQN algorithm and Regret learning user association algorithm in our proposed framework are both replaced by the GA. Moreover, we evaluate and analyze our proposed framework by comparing it to these different scenarios and algorithms ensuring they have the same total capacities and simulation parameters. The rest of NR, NR-U, WLAN, and DDQN network parameters are summarized in Table 3^25,34^

### Results and discussion

To start, we evaluate the effectiveness of our proposed framework by deploying multi-RAT SCs utilizing NR-U technology features on the unlicensed band. We compared the performance of this deployment to a traditional NR-only model, an NR-WiFi model, and an LWA model, as shown in Fig. 2.

Figure 2 illustrates how users’ average satisfaction varies with the increasing number of users in the network ($U=40$ to $U=80$). The users’ average satisfaction is defined as the total satisfaction of all users across slices and SCs, divided by the total number of users in the network (*U*). The performance of the proposed model is evaluated and compared with the three baseline models, where the users’ request ($R_j$) is set to 0.5. This value of $R_j$ indicates that an equal proportion of users request eMBB and URLLC services.

It can be noted that our proposed framework outperforms the three compared models (NR-only, NR-WiFi, and LWA). Compared to the other models, our framework guarantees to satisfy each user’s service request by finding the optimum slice resource allocation ratio and selecting the optimum numerology value in both the licensed and unlicensed bands, exploiting all NR features in the unlicensed band. In the NR-only model, all the base stations operate solely under NR, which explains the huge degradation in users’ average satisfaction compared to other baseline approaches due to NR limited resources.

In contrast, in the NR-WiFi model, users’ average satisfaction is enhanced compared to the NR-only model, as the presence of the unlicensed band (WiFi) alleviates the traffic load and supports the limited capacity of NR. However, the NR-WiFi model shows a decrease in user average satisfaction with the increase in the number of users, reaching U=80. This decline can be attributed to congestion in both LTE and WiFi base stations, which become overwhelmed as the number of users increases.

**Fig. 4:**
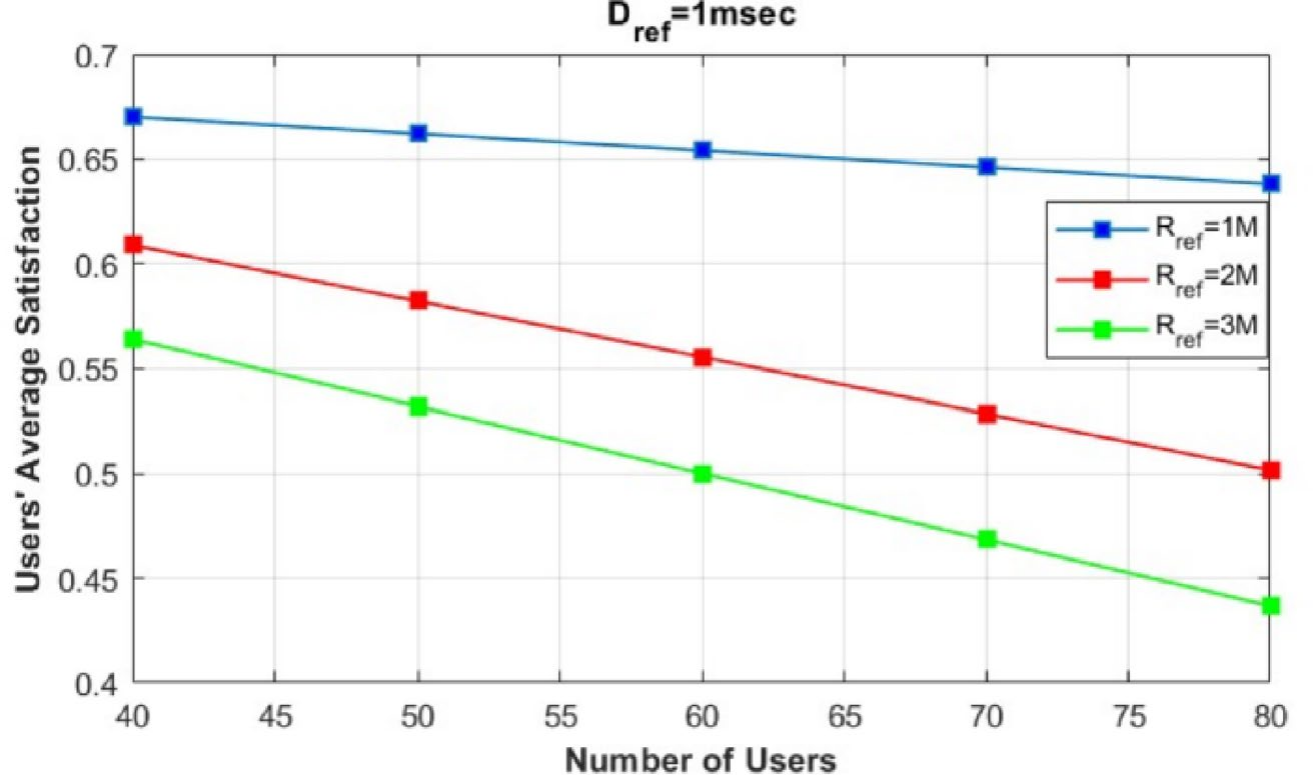
Evaluation of Users’ average satisfaction of our proposed model with different Rref values.

**Fig. 5:**
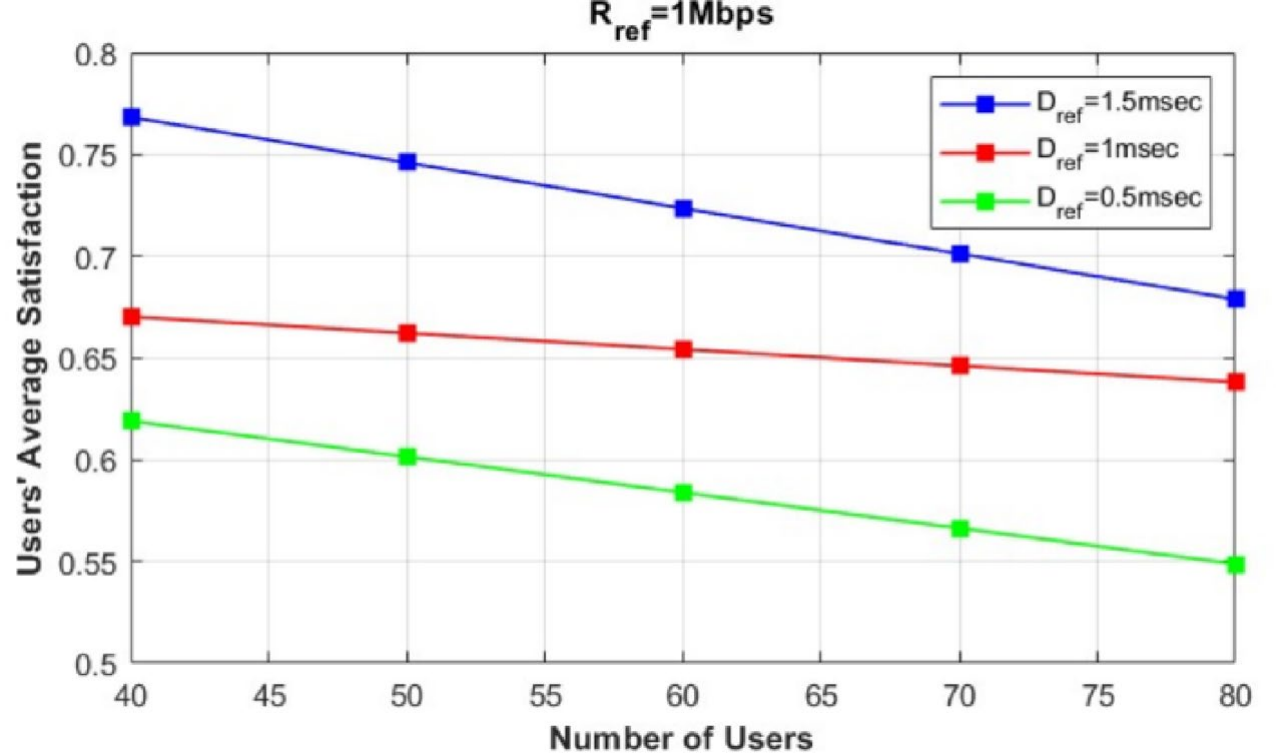
Evaluation of users’ average satisfaction of our proposed model with different Dref values.

In LWA model, an enhancement is shown compared to NR-only model and NR-WiFi mode. As the LWA model offers three modes of operation, utilizing both the licensed and unlicensed band in addition to aggregation mode, which can be very effective in congested demands. However, it limited to applying network slicing and scalable numerology to only NR licensed band, which won’t efficiently guarantee each user satisfaction especially on unlicensed band.

Additionally, Fig. 3 analyzes the variation in users’ average satisfaction as the number of users in the network increases ($U=40$ to $U=80$) under different values of the users’ request parameter ($R_j$). Specifically, when $R_j=0.2$, 20% of the total users request eMBB service, while the remaining 80% request URLLC service. Similarly, when $R_j=0.8$, 80% of the total users request eMBB service, while the remaining 20% request URLLC service.

We compared our proposed model with the LWA model^20^, which utilizes network slicing and scalable numerology techniques in a HetNet architecture while also exploiting the unlicensed band to aggregate both LTE and WiFi, forming three modes of transmission. This approach can enhance users’ satisfaction, particularly under high-demand conditions. However, as shown in Fig. 3, our proposed model outperforms the LWA model for both values of $R_j$. We attribute this improvement to the application of network slicing and scalable numerology in the unlicensed band, exploiting the NR-U features. Moreover, our proposed NR-U model is characterized by the CA mode, which guarantees the fulfillment of each user’s service request requirements, especially during high demand.

Additionally, to demonstrate the effectiveness of our proposed framework, Fig. 4 illustrates the effect of varying the $R_{\text {ref}}$ values on users’ average satisfaction. In this figure, we simulate our proposed framework by varying $R_{\text {ref}}$ from 1 Mbps to 3 Mbps and analyze how this variation impacts users’ average satisfaction. We consider an average rate request ($R_j = 0.5$), where the number of users requesting eMBB service is equal to the number of users requesting URLLC service, while varying the total number of users from $U=40$ to $U=80$. As shown, users’ average satisfaction generally decreases with increasing $R_{\text {ref}}$ values and number of users. Nevertheless, our proposed framework is able to maintain an average satisfaction level between 55 and 76%.

In contrast, Fig. 5 shows the effect of changing the $D_{\text {ref}}$ values on users’ average satisfaction. In this figure, we evaluate our proposed framework by varying $D_{\text {ref}}$ from 0.5 ms to 1.5 ms and analyze how this impacts users’ average satisfaction. We consider an average rate request ($R_j = 0.5$), where the number of users requesting eMBB service is equal to the number of users requesting URLLC service, while varying the total number of users from $U=40$ to $U=80$. As shown, users’ average satisfaction generally decreases as $D_{\text {ref}}$ decreases. However, in our proposed model, users’ average satisfaction remains at 55% even when the delay request is as low as 0.5 ms, which is below the requirements of most URLLC standard services.

**Table 4:** Variation of $\alpha _j$ values across different base stations.

$R_j/\alpha _j$	MBS	NR SC	NR (CA)	NR-U (CA)	NR-U
0.2	42.15	26.2	20.6	94.25	80.65
0.5	77.45	42.35	58.25	99.3	74.55
0.8	79.4	76.3	80.55	99.65	94.65

**Table 5:** Variation of $\omega _j$ values across different base stations.

$R_j/\omega _j$	MBS	NR SC	NR (CA)	NR-U (CA)	NR-U
0.2	3	3	3	1	2
0.5	2	3	2	1	1
0.8	1	3	3	1	1

It may be concluded that our proposed framework has the capability to achieve and maintain users’ satisfaction regardless of the type of service requested. This is accomplished by leveraging NR-U technology in combination with NR scalable numerology and network slicing in both licensed and unlicensed bands. By proposing such a framework, we can increase capacity and improve the overall user experience for both high data rate and low-latency services.

**Fig. 6:**
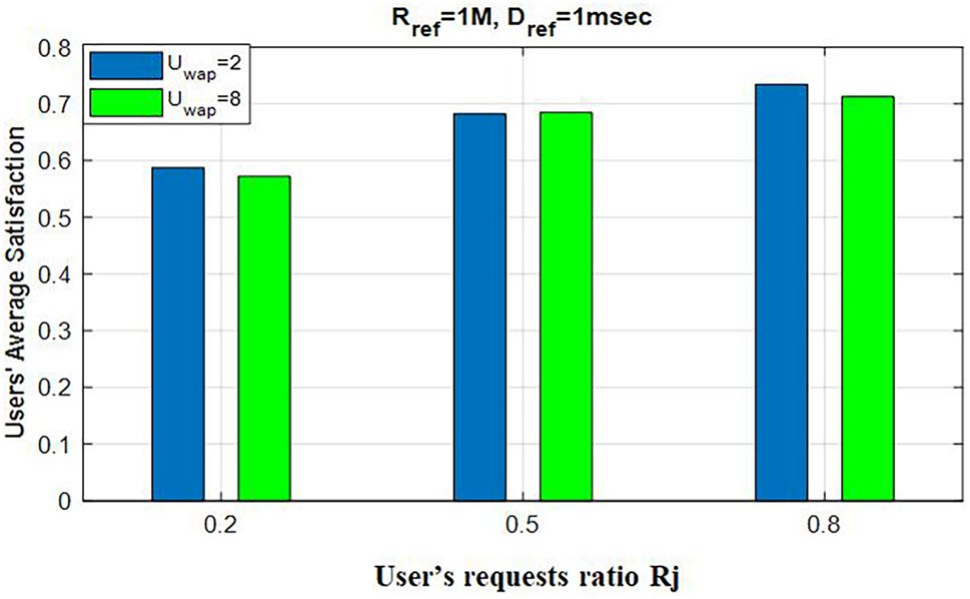
Evaluation of users’ average satisfaction varying users requests ratio $R_j$ values with different $U_{\text {wap}}$.

Moreover, we analyze the effect of varying the number of coexisting WiFi users on each channel to study the contention process between NR-U users and WiFi users and its impact on users’ satisfaction. Figure 6 presents the evaluation of users’ average satisfaction with U=50 users while varying $U_{\text {wap}}$. We compare a minimum number of WiFi users at $U_{\text {wap}}=2$ with $U_{\text {wap}}=8$ users per channel, alongside changes in $R_j$. As shown in Fig. 6, although the increase in the number of WiFi users leads to degradation in users’ average satisfaction due to contention and the LBT process, the degradation gap between the two different $U_{\text {wap}}$ values remains small and manageable for each $R_j$ value. This demonstrates the effectiveness of our proposed model, which strikes a balance between the different users’ service requests while considering the coexistence of WiFi users. In addition, our proposed model deploys the regret learning game, which effectively can guarantee each user will connect to the base station that fulfills their SLA.

**Fig. 7:**
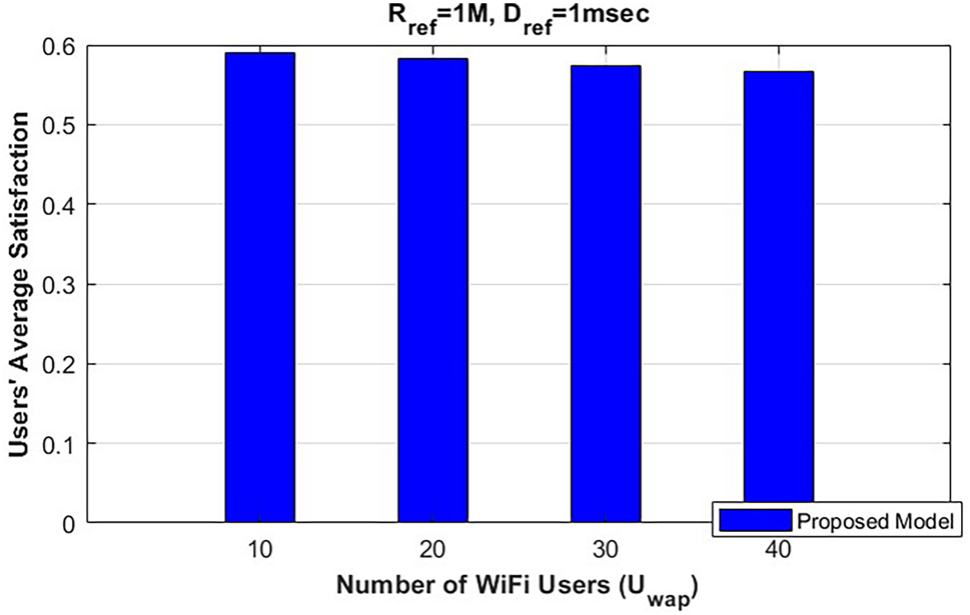
Evaluation of users’ average satisfaction in a dense WiFi co-exist users environment ranging from $U_{\text {wap}}$=10 to $U_{\text {wap}}$= 40.

In addition, Fig. 7 examines the effect of increasing the number of coexisting Wi-Fi users, representing a dense environment scenario, and how this impacts users’ average satisfaction. Figure 7 evaluates users’ average satisfaction with NR-U ($U = 50$) users while increasing the number of WiFi users from $U_{\text {wap}}$=10 to $U_{\text {wap}}$= 40. As shown in Fig. 7, increasing the number of WiFi users leads to a degradation in users’ average satisfaction; however, the degradation is very small, with a gap of approximately 0.02. This demonstrates the effectiveness of our proposed model, which dynamically adapts the slice ratio and scalable numerology values to maintain each user’s satisfaction, in addition to the different transmission modes, including the CA mode that can utilize both the licensed and unlicensed bands.

In addition, Tables 4, 5 represents the variation in $\alpha _j$ and $\omega _j$ values per slice and per base station, to satisfy each user’s service request requirement. As $\alpha _j$ represents the eMBB slice resources ratio, then $1-\alpha _j$ represents the URLLC slice resources ratio. In contrast, the values of $\omega _j$ shown in Table define the fluctuation in scalable numerology values based on users’ service requests.

**Fig. 8:**
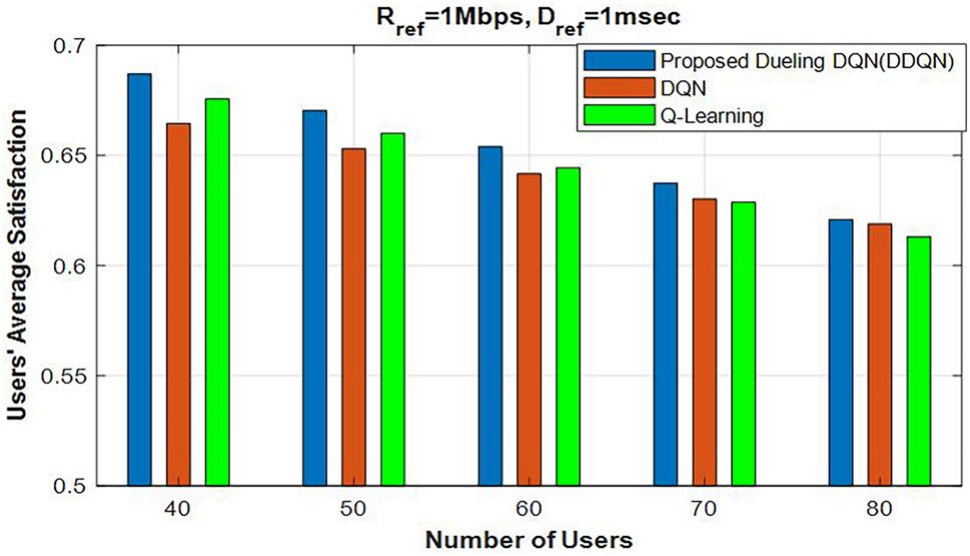
Evaluation of users’ average satisfaction of our proposed model using DDQN compared to DQN and Q-Learning approaches.

Moreover, we conducted a comparison between our proposed DDQN algorithm and two other RL-based algorithms, namely Q-Learning and DQN, as shown in Fig. 8. We analyzed the variations in achieving users’ average satisfaction as the number of users in the network increased. As shown in Fig. 8, the proposed DDQN algorithm outperformed the other two RL-based algorithms, since DDQN, as developed in 2016, improves the system’s convergence speed compared to other learning algorithms. This innovative neural network architecture estimates state values and action advantages separately using two streams of fully connected layers, rather than relying only on the action-value function (Q function). The outputs are combined at the final layer; this approach is effective because, in many cases, the value of certain actions does not influence outcomes. As a result, the DDQN algorithm provides more accurate state value estimates, improving both convergence speed and stability. In contrast, from the perspective of time complexity, the Q-Learning and DQN algorithms are simpler, yet they are limited, especially in large-scale systems. The Q-Learning algorithm can easily converge after *I* iterations and *T* slot times. Therefore, the computational complexity of the Q-Learning algorithm can be expressed as: $\ O(IT)$ while the computational complexity of the DQN algorithm relies on more parameters^35^, including, the number of iterations *I*, the slot time *T*, the number of hidden layers *L*, in the DQN neural network, the number of neurons $\ h^{2}$ in each layer and the size of the state-action space $\ V$. Therefore, the time complexity of DQN to converge can be expressed as $\ O\!\left( IT \left( V + Lh^{2} \right) \right)$

In addition, the computational complexity of the DDQN algorithm can be expressed by^27^ $O(N_0^{neu} N_1^{neu}+\sum _{(\iota =1)}^{L}N_\iota ^{neu} N_{(\iota +1)}^{neu})$. Where, *L*, $N_0^{neu}$, and $N_\iota ^{neu}$ denote the number of layers, the size of the input layer, and the size of layer $\iota$, respectively.

**Fig. 9:**
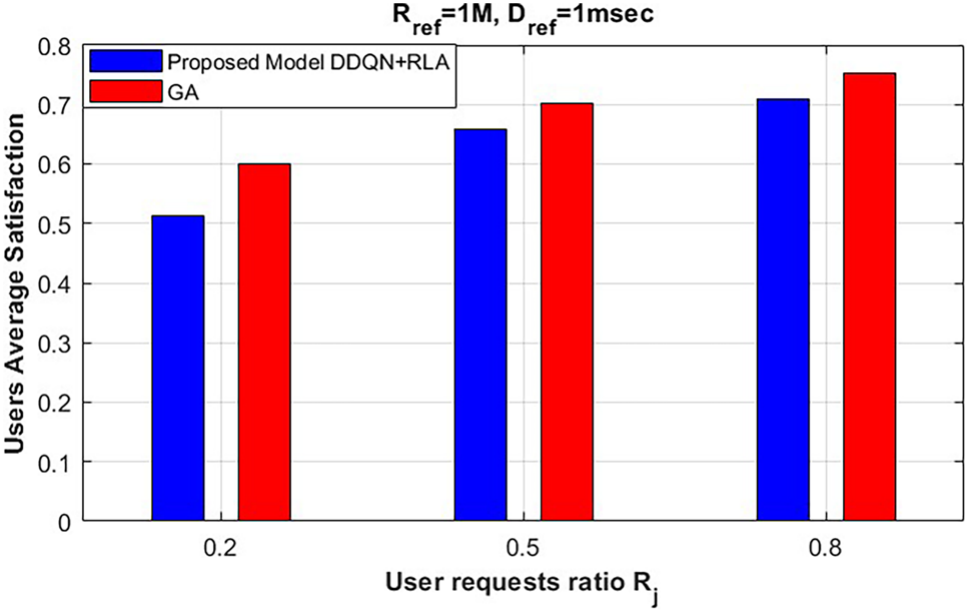
Evaluation of users’ average satisfaction of our proposed model compared to GA, varying users’ requests ratio $R_j$.

Moreover, our proposed model is compared to the Heuristic–Genetic Algorithm (GA) as shown in Fig. 9, in terms of users’ average satisfaction. In this figure, we evaluate our proposed framework by varying $R_j=0.2$ to $R_j = 0.8$, with $U=40$, Our proposed framework has demonstrated notable success in achieving high average user satisfaction values, reaching approximately a slightly average difference in three different values of $R_j$ by 8%, comparable to the results obtained by GA.

In addition, the advantage of our proposed model is defined in the integration between the NR licensed and unlicensed bands, which results in alleviating traffic congestion, improving network capacity, and helping mitigate the diversity in users’ service requests. In addition, deploying network slicing and scalable numerology techniques in NR-U can improve users’ satisfaction and maintain each user’s QoS. In contrast, our limitation is that each user’s service request has different requirements; therefore, deploying a single numerology value for each slice may limit the guarantee of each user’s SLA. For example, a URLLC slice can include different traffic classes, each with distinct delay requirements, which may require different numerology values. This limitation remains an open challenge that can be studied and investigated in future work by assuming a specific numerology for each user request. Moreover, the learning-based optimization approach introduces computational complexity, particularly when the number of slices increases. As the network scales, the training time and convergence of the learning algorithms may require more processing or distributed learning strategies. This scalability challenge can be further investigated in future work.

## Conclusion

This study presents a dynamic radio resource allocation scheme for eMBB and URLLC slices within a Multi-RAT HetNet architecture. The proposed framework leverages network slicing and the 6G scalable numerology technique in both NR and NR-U to overcome the capacity limitations of NR networks while considering Wi-Fi coexisting users. A DDQN algorithm jointly with the Regret Learning Algorithm is proposed to efficiently allocate radio resources for each slice, determine the optimal numerology value, and solve the user association problem. To assess the effectiveness of this approach, we compare its performance against various alternative scenarios, differing in both network architecture and the algorithms employed. Simulation results demonstrate that the proposed model achieves significant enhancement in users’ satisfaction compared to other baseline approaches as NR-only model, NR-WiFi model and LWA model, showing the effectiveness in utilizing the NR in the unlicensed band in terms of coverage, capacity, and maintaining each user’s QoS.

## Methods

Our Propsed model is evaluated and simulated using Matlab R2022a, For the traiing Adam Optimizer is used with the follwoing values: Learning rate with $2\times 10^{-8}$^36^ and 0.0001 $L_2$ regularization factor. The DDQN target network is updated every 10 steps, with a replay buffer size of 100,000. An episode terminates once the highest-reward target is found or when the maximum episode limit is reached. Regret learning stops upon reaching its predefined maximum iterations^37^.

## Data Availability

The source files/datasets used and/or analyzed during the current study available from the corresponding author on reasonable request.
